# Molecular and functional characterization of somatostatin-type signalling in a deuterostome invertebrate

**DOI:** 10.1098/rsob.200172

**Published:** 2020-09-09

**Authors:** Ya Zhang, Luis Alfonso Yañez Guerra, Michaela Egertová, Cleidiane G. Zampronio, Alexandra M. Jones, Maurice R. Elphick

**Affiliations:** 1 School of Biological and Chemical Sciences, Queen Mary University of London, London E1 4NS, UK; 2 School of Life Sciences and Proteomics Research Technology Platform, University of Warwick, Coventry CV4 7AL, UK

**Keywords:** neuropeptide, evolution, receptor, somatostatin, muscle, starfish

## Abstract

Somatostatin (SS) and allatostatin-C (ASTC) are structurally and evolutionarily related neuropeptides that act as inhibitory regulators of physiological processes in mammals and insects, respectively. Here, we report the first molecular and functional characterization of SS/ASTC-type signalling in a deuterostome invertebrate—the starfish *Asterias rubens* (phylum Echinodermata). Two SS/ASTC-type precursors were identified in *A. rubens* (ArSSP1 and ArSSP2) and the structures of neuropeptides derived from these proteins (ArSS1 and ArSS2) were analysed using mass spectrometry. Pharmacological characterization of three cloned *A. rubens* SS/ASTC-type receptors (ArSSR1–3) revealed that ArSS2, but not ArSS1, acts as a ligand for all three receptors. Analysis of ArSS2 expression in *A. rubens* using mRNA *in situ* hybridization and immunohistochemistry revealed stained cells/fibres in the central nervous system, the digestive system (e.g. cardiac stomach) and the body wall and its appendages (e.g. tube feet). Furthermore, *in vitro* pharmacological tests revealed that ArSS2 causes dose-dependent relaxation of tube foot and cardiac stomach preparations, while injection of ArSS2 *in vivo* causes partial eversion of the cardiac stomach. Our findings provide new insights into the molecular evolution of SS/ASTC-type signalling in the animal kingdom and reveal an ancient role of SS-type neuropeptides as inhibitory regulators of muscle contractility.

## 1. Introduction

Somatostatin (SS) was first identified as a constituent of rat hypothalamus that inhibits growth hormone release [1] and a constituent of pigeon pancreatic islets that inhibits insulin release [2]. Purification and sequencing of SS revealed that it is a tetradecapeptide with a disulfide bridge linking cysteine residues at positions 3 and 14 (SS14) [3]. Subsequently, an N-terminally extended isoform (SS28) was identified [4], with both SS14 and SS28 being derived from the same precursor protein [5]. Five G-protein-coupled receptors, SSTR1, SSTR2, SSTR3, SSTR4 and SSTR5, mediate effects of SS in mammals as a regulator of a variety of physiological processes [6,7]. In addition to its effects as an inhibitor of hormone release, other actions of SS in mammals include inhibition of pancreatic exocrine secretion [8], regulation of gut motility [8,9] and neuromodulation in the central nervous system [10,11]. Behavioural effects of intracerebroventricular administration of SS in mammals include stimulation of food intake and drinking [12,13] and anxiolysis [14].

The neuropeptides cortistatin (CST), urotensin II (UII) and urotensin II-related peptide (URP) share sequence similarity with SS, including the presence of two cysteines that form a disulfide bridge, indicating that these peptides have a common evolutionary origin [15]. Accordingly, analysis of the phylogenetic distribution of these neuropeptides in vertebrates indicates that tandem gene duplication gave rise to genes encoding an SS/CST-type peptide and a UII/URP-type peptide. Then two or three rounds of genome duplication followed by differential gene loss gave rise to the variable numbers of genes encoding SS/UII-type peptides in different vertebrate lineages, ranging from four genes in humans (SS, CST, UII, URP) to as many as eleven genes in the zebrafish *Danio rerio*. Likewise, variable numbers of receptors for SS/UII-type peptides are found in different vertebrate lineages [15]; in humans, for example, in addition to five receptors that mediate effects of SS and CST, there is a single receptor that mediates effects of UII and URP [16,17]. Other GPCRs that are closely related to SS receptors have also been identified in vertebrates and deorphanization has revealed that melanin-concentrating hormone (MCH) acts as a ligand for these receptors. Consistent with this finding, MCH shares structural similarity with SS in having two cysteine residues that form a disulfide bridge in the mature peptide [18,19].

Phylogenetic analysis of neuropeptide signalling throughout the animal kingdom has revealed relationships between neuropeptides in vertebrates and invertebrates, with the evolutionary origin of at least 30 neuropeptide systems being traced back to the common ancestor of the Bilateria [20]. Accordingly, allatostatin-C (ASTC) type neuropeptides and their receptors in protostome invertebrates are recognized as homologues of vertebrate SS-type peptides and their receptors, respectively [21,22]. ASTC was first identified in the insect *Manduca sexta* on account of its inhibitory effect on juvenile hormone biosynthesis, with structural characterization revealing that it is a 15-residue peptide with a disulfide bridge formed by two cysteine residues [23]. Subsequently, ASTC-type peptides were identified in other insect species and the receptors that mediate effects of ASTC were identified in *Drosophila melanogaster*. Furthermore, discovery of these receptors revealed that ASTC-type receptors in insects are closely related to SS-type receptors and opioid-type receptors in vertebrates [21]. Subsequently, a paralogue of ASTC named allatostatin double C (ASTCC) was identified in *D. melanogaster* [24] and ASTC-like peptides were identified in other protostome invertebrates, including molluscs [25–28] and annelids [29].

Investigation of the physiological roles of ASTC-type signalling in *D. melanogaster* has revealed roles in cardioinhibition [30], anti-nociception, immunosuppression [31] and regulation of circadian activity [32]. Furthermore, investigation of ASTC-type neuropeptide function in other arthropods has revealed roles in inhibition of gut myoactivity and suppression of feeding/growth in the tomato moth *Lacanobia oleracea* [33,34] and in the pea aphid *Acyrthosiphon pisum* [35], modulation of learning in bees [36,37], inhibitory regulation of post-prandial heart and gut activity in the blood-sucking bug *Rhodnius prolixus* [38], regulation of cardiac and foregut activity in the lobster *Homarus americanus* and the crab *Cancer borealis* [39–43], and inhibition of ovarian development in the mud crab *Scylla paramamosain* [44]. To the best of our knowledge, however, nothing is known about the physiological roles of ASTC-type neuropeptides in other protostomes (e.g. molluscs, annelids).

Important insights into the evolution and comparative physiology of neuropeptide signalling systems have been obtained from research on deuterostome invertebrates (e.g. urochordates, echinoderms), which are more closely related to vertebrates than protostome invertebrates [45–47]. Accordingly, by analysing transcriptome/genome sequence data from echinoderms, we recently reported the discovery of precursor proteins that comprise peptides that share sequence similarity with SS/ASTC-type neuropeptides [48,49]. However, the receptors that mediate the effects of echinoderm SS/ASTC-like peptides have not been identified. Furthermore, nothing is known about the expression and physiological roles of SS/ASTC-like neuropeptides in echinoderms. Therefore, here we used the starfish *A. rubens* as an experimental system to address this issue. This is the first study to report the molecular and functional characterization of SS-type signalling in a deuterostome invertebrate.

## 2. Material and methods

### 2.1. Animals

Starfish (*A. rubens*) were collected at low tide near Margate (Kent, UK) or were obtained from a fisherman based in Whitstable (Kent, UK), and then transported to an aquarium located in the School of Biological and Chemical Sciences at Queen Mary University of London. Here, the animals were maintained in circulating artificial seawater (Premium Reef-Salt, Tropical Marine Centre, Rickmansworth, UK) at a salinity of 32‰ and at a temperature of approximately 12°C under a 12 h/12 h light/dark cycle. The starfish were fed on mussels (*Mytilus edulis*) that were also collected near Margate (Kent, UK) at low tide.

### 2.2. Cloning and sequencing of cDNAs encoding *A. rubens* precursors of SS-like peptides

Two transcripts encoding precursors of SS-like peptides were identified by analysis of *A. rubens* neural transcriptome sequence data—ArSSP1 (GenBank: KT601708) and ArSSP2 (GenBank: MN257487). The sequence of ArSSP1 has been reported previously [48]. The sequence of ArSSP2 is reported here for the first time and was identified on account of its similarity to precursors of SS-like peptides that have been identified in other echinoderms [49]. Informed by the assembled transcript sequences of ArSSP1 and ArSSP2, cDNAs comprising the complete open reading frame of ArSSP1 and ArSSP2 were amplified from *A. rubens* radial nerve cord cDNA by PCR using specific primers (electronic supplementary material, table S1) and Q5 High-fidelity DNA polymerase (NEB, Hitchin, UK; cat. no. M0491S), cloned into pBluescript II SK+ vector (Agilent Technologies, Santa Clara, USA; cat. no. 212205) and then sequenced (TubeSeq service; Eurofins Genomics, Ebersberg, Germany).

### 2.3. Structural characterization of the ArSSP1- and ArSSP2-derived neuropeptides ArSS1 and ArSS2 in extracts of *A. rubens* radial nerve cords using mass spectrometry

Extracts of *A. rubens* radial nerve cords were prepared and analysed using mass spectrometry (NanoLC-ESI-MS/MS) as described in detail previously for *A. rubens* relaxin-like gonad-stimulating peptide, a neuropeptide that contains disulfide bridges [50]. Because both ArSS1 and ArSS2 are predicted to contain a disulfide bridge, samples of extracts were treated with dithiothreitol (DTT) for reduction to break disulfide bridges and then were treated with iodoacetamide to alkylate cysteine residues. For comparison, samples of extracts without reduction and alkylation were also analysed.

### 2.4. Sequence alignment of echinoderm SS-like peptides with SS-related peptides in other taxa

The amino acid sequences of ArSS1 and ArSS2 and related peptides from other echinoderms were aligned with the sequences of SS-related neuropeptides from a variety of bilaterian species (see electronic supplementary material, table S2 for a list of the sequences). This was accomplished using MAFFT 7, with the iterative refinement method set to L-INS-i and scoring matrix for amino acid sequences set to BLOSUM62, ensuring an optimal alignment. BOXSHADE software (https://embnet.vital-it.ch/software/BOX_form.html) was used to highlight residues that are identical or are conservative substitutions in at least 70% of the aligned sequences.

### 2.5. Comparison of the exon/intron structure of genes encoding SS-like peptides in echinoderms and other taxa

The sequences of transcripts and genes encoding precursors of SS-related peptides were obtained from the NCBI GenBank database (see electronic supplementary material, table S3 for a list of the transcript and gene sequences used). The online tool Splign [51] was used to determine gene structure and IBS 1.0 [52] was used to generate diagrams of gene structures.

### 2.6. Identification of candidate SS-type receptors in *A. rubens* and phylogenetic analysis of their relationship with related receptors in other bilaterians

BLAST analysis of an *A. rubens* neural transcriptome sequence database [48,53] was performed to identify transcripts encoding candidate SS-type receptors. To accomplish this, SequenceServer [54] was used, submitting sequences of SS-type receptors from *Homo sapiens* (GenBank; NP_001040.1, NP_001041.1, NP_001042.1, NP_001043.2, NP_001044.1), ASTC-type receptors from *D. melanogaster* (GenBank; NP_001027135.2, NP_649040.2), MCH-type receptors from *H. sapiens* (GenBank; NP_005288.3, NP_001035269.1) as query sequences. Five transcripts were identified as the top hits in all BLAST searches and the five encoded protein sequences were analysed using Protter v. 1.0 to determine the locations of predicted membrane-spanning domains and other structural features [55]. Using BLAST, homologues of the *A. rubens* receptor proteins were identified in other echinoderms, including the starfish *Acanthaster planci*, the brittle star *Ophionotus victoriae,* the sea urchin *Strongylocentrotus purpuratus* and the sea cucumber *Apostichopus japonicus*.

Relationships of the five *A. rubens* receptors with closely related receptors in other taxa types were investigated by alignment with SS/ASTC/opioid-type, MCH-type and urotensin II-type receptors, using galanin-type and kisspeptin-type receptors as outgroups (see electronic supplementary material, table S4 for a list of sequences, which were obtained from the NCBI GenBank database). Alignments were generated using the MUSCLE algorithm in MEGA 7 (iterative, 10 iterations, UPGMB as clustering method) [56,57]. Next the alignment was manually trimmed N-terminally and C-terminally to produce an alignment of 396 residues (including gaps) spanning from the beginning of the predicted first transmembrane domain to end of the seventh transmembrane domain. Then the maximum-likelihood method was used to generate a phylogenetic tree using W-IQ-tree online version 1.0 (1000 bootstrap replicates, LG + F+I + G4 substitution model) [57] and a graphic of the phylogenetic tree was produced using FigTree v. 1.4.4 [58]. A second alignment was manually trimmed in the regions where only one sequence did not align with any other sequence. This version had less trimming of the sequences compared with the first alignment and included a total of 523 residues (including gaps) spanning from the first to the seventh transmembrane domains; a phylogenetic tree was then generated as described above for the first alignment (see electronic supplementary material, figure S5).

### 2.7. Pharmacological characterization of *A. rubens* SS-type receptors

Three cDNAs encoding SS-type receptors were amplified from *A. rubens* radial nerve cord total cDNA by PCR, using specific primers (see electronic supplementary material, table S1) and Q5 High-fidelity DNA polymerase (NEB, Hitchin, UK; cat. no. M0491S). PCR products comprising the complete open reading frame with a partial Kozak consensus sequence (ACC) before the ATG start codon [59] were phosphorylated using T4 polynucleotide kinase for 2 h (Thermo Fisher Scientific, Oxford, UK; cat. no. EK0031) and then purified using a QIAquick gel extraction kit (Qiagen, Hilden, Germany; cat. no. 28704). Before blunt cloning PCR products into the expression vector pcDNA3.1+ (Invitrogen, Oxford, UK; cat. no. v. 790–20) using T4 DNA ligase (NEB, Hitchin, UK; cat. no. M0202S), the pcDNA3.1+ vector was cut with the restriction enzyme *EcoRV-*HF (NEB, Hitchin, UK; cat. no. R3195T) and processed with Shrimp Alkaline Phosphatase (NEB, Hitchin, UK; cat. no. M0371S) to avoid vector self-ligation. Successful cloning of cDNAs was confirmed by PCR and sequencing (TubeSeq service, Eurofins Genomics, Ebersberg, Germany).

In order to characterize the candidate SS-type receptors, a G-protein-dependent receptor activation assay was employed [60]. Chinese hamster ovary (CHO)-K1 cells stably overexpressing the calcium-sensitive bioluminescent reporter GFP-aequorin fusion protein (G5A) were used as a heterologous expression system [61]. Plasmids containing *A. rubens* SS-type receptor cDNAs were co-transfected with the promiscuous human Gα16, which is widely used for receptor deorphanization assays [62]. Firstly, CHO-K1 cells were cultured at 37°C with 5% carbon dioxide (CO_2_) in a culture medium comprising DMEM/F12, 10% fetal bovine serum, Antibiotic-Antimycotic 1x, and 200 µg ml^−1^ of Geneticin G418 sulfate (all purchased from Thermo Fisher Scientific, Oxford, UK; cat. no. 11039047, 10082147, 15240062 and 10131035, respectively). Upon reaching confluency of approximately 80%, cells were co-transfected with plasmids containing an *A. rubens* SS-type receptor cDNA and Gα16 using Lipofectamine 3000 (Thermo Fisher Scientific, Oxford, UK; cat. no. L3000008). Cells transfected with the empty pcDNA3.1+ vector were used as a negative control. A detailed description of the method used for this assay has been described for deorphanization of luqin-type receptors in *A. rubens* [63]. Forty-eight hours after transfection, cells were detached by using PBS buffer pH 7.4 (Thermo Fisher Scientific, Oxford, UK; cat. no. 10010023) supplemented with a final concentration of 5 mM EDTA pH 8.0 (Thermo Fisher Scientific, Oxford, UK; cat. no. 15575020). Cells were washed with DMEM/F12 buffer and then incubated with 1 mM coelenterazine-H (Thermo Fisher Scientific, Oxford, UK; cat. no. C6780) at room temperature for 3 h. During the incubation time, synthetic ArSS1 or ArSS2 (custom synthesized by Peptide Protein Research Ltd; Fareham, UK) diluted in DMEM/F12 medium at concentrations ranging from 10^−21^ to 10^−4^ M were pipetted into clear bottom 96-well plates sequentially (Sigma-Aldrich, Gillingham, UK; cat. no. CLS3603-48EA). After 3 h incubation, a fixed volume of cells was injected sequentially into each well containing different concentrations of peptides using a FLUOstar Omega Plate Reader (BMG LABTECH, Ortenberg, Germany). Luminescence values were recorded over a 35 s period after injection. The total luminescence of each well over the 35 s period was calculated and normalized to the maximum value obtained in each experiment (100% activation) and to the value obtained with the vehicle media (0% activation). Dose–response curves were fitted with a four-parameter curve using Prism 6 (GraphPad, La Jolla, CA, USA) based on data collected from at least three independent transfections and three repeats per experiment. EC_50_ values were calculated from dose–response curves using Prism 6. The luminescence responses triggered by 10^−5^ M ArSS1, 10^−5^ M ArSS2 and vehicle media were analysed by one-way ANOVA using Bonferroni's multiple comparisons *post hoc* test in Prism 6.

### 2.8. Localization of ArSSP2 expression in *A. rubens* using mRNA *in situ* hybridization

ArSSP2 cDNA was amplified by PCR from a pBluescript II SK+ plasmid containing the cloned ArSSP2 cDNA using standard M13 primers (see electronic supplementary material, table S1) and Q5 High-fidelity DNA polymerase (NEB, Hitchin, UK; cat. no. M0491S). Then PCR products were purified using the QIAquick gel extraction kit (Qiagen, Hilden, Germany; cat. No. 28704). Antisense and sense probes were generated by incubating the purified PCR product with digoxigenin (DIG)-labelled nucleotide triphosphate mix (Roche, Hertfordshire, UK; cat. no. 11277073910) supplemented with dithiothreitol (Promega, Hampshire, UK; cat. no. P1171), placental ribonuclease inhibitor (NEB, Hitchin, UK; cat. no. M0307S), T3 or T7 RNA polymerase with transcription buffer (NEB, Hitchin, UK, cat. no. M0251S) at 37°C for 2 h. The DNA templates were digested with RNAse-free DNAse (NEB, Hitchin, UK; cat. no. M0303S) at 37°C for 30 min and then the labelled RNA probes were precipitated and stored in 25% formamide made up in 2× saline-sodium citrate (SSC) buffer at −20°C.

Small (diameter 4–8 cm) or medium (diameter 8–12 cm) sized adult specimens of *A. rubens* were fixed in 4% paraformaldehyde (PFA; Sigma-Aldrich, Gillingham, UK) for at least 24 h at 4°C. The specimens were washed in autoclaved distilled water and then dissected to separate arms from the central disc region. Morse's solution (10% sodium citrate and 20% formic acid dissolved in autoclaved water) was used to decalcify the arms and central disc of the starfish at room temperature for at least 3 and 8 h, respectively, with the Morse's solution replenished every 2 h. Then the decalcified tissues were washed in autoclaved water and dehydrated sequentially in 50, 70, 90 and 100% ethanol. After clearing in xylene (3 × 5 min), the tissues were transferred to melted filtered paraffin wax (three changes) at approximately 60°C, and then embedded in wax at room temperature. Sections of arms/discs (14 µm) were prepared using a microtome (RM 2145, Leica Microsystems, Milton Keynes, UK), mounted over water on Poly-l-lysine coated microscope slides (Polysine; VWR, Lutterworth, UK) and then left to dry overnight at room temperature.

Prior to mRNA *in situ* hybridization, xylene was used to remove the paraffin wax (three changes) and then an ethanol series (100, 90, 70, 50, 30%) followed by PBS was used to rehydrate the sections. Next the sections were fixed in 4% PFA/PBS, followed by washing in PBS/0.1% Tween-20 (PBST). After incubation with Proteinase K (Qiagen, Hilden, Germany) at 37°C, the sections were fixed in 4% PFA/PBS and washed again in PBST. To reduce non-specific background staining, sections were acetylated (1.3% triethanolamine, pH 7–8; 0.25% acetic anhydride; 0.18% acetic acid; (VWR Chemicals, Lutterworth, UK) made up in distilled water) and then following washing in PBST and 5 x SSC solution, the sections were pre-hybridized in hybridization buffer (50% formamide (Amresco, Solon, USA); 5 x SSC; 500 µg ml^−1^ yeast total RNA (Sigma-Aldrich, Gillingham, UK); 50 µg ml^−1^ heparin (Sigma-Aldrich, Gillingham, UK); 0.1% Tween-20 in distilled water) for 2 h. Antisense or sense probes (500 ng m^−1^) were denatured at 80°C for 2 min and then approximately 100 µl was added to each slide, which was then covered with Parafilm (Bemis, Terre Haute, USA) to reduce evaporation. The slides were placed in humidified chambers and incubated at 60°C overnight. On the second day, the slides were washed in 0.2 x SSC, equilibrated in Buffer B1 (10 mM Tris–HCl (pH 7.5); 150 mM NaCl in autoclaved water) and blocked in 5% goat serum/B1 solution for 2 h. Then the slides were incubated with alkaline phosphatase (AP)-conjugated anti-DIG antibodies (1 : 3000; Roche, Hertfordshire, UK) in 2.5% goat serum/B1 solution at 4^°^C overnight. On the third day, the slides were washed in B1 Buffer and B3 Buffer (100 mM Tris–HCl (pH 9.5); 100 mM NaCl, 50 mM MgCl_2_ in autoclaved water) and then incubated with 500 µl of AP substrate containing 4.5 µl ml^−1^ of nitro-blue tetrazolium chloride (NBT; Amresco, Solon, USA) stock solution (75 mg ml^−1^ in 70% dimethylformamide) and 3.5 µl ml^−1^ of 5-bromo-4-chloro-30-indolyphosphate *p*-toluidine (BCIP; Panreac AppliChem, Darmstadt, Germany) stock solution (50 mg ml^−1^ in 70% dimethylformamide) in dark humidified chambers. When strong staining was observed, the slides were washed in autoclaved water, dried on a hot plate, briefly washed in 100% ethanol (2 × 10 s) and then cleared in Histo-Clear (National Diagnostics, Nottingham, UK). Lastly, the slides were mounted with a coverslip using HistoMount (National Diagnostics, Nottingham, UK). Images of stained sections were captured using a QIClich CCD Colour Camera (Qimaging, Birmingham, UK) linked to a DMRAI light microscope (Leica Microsystems, Milton Keynes, UK) and Volocity v. 6.3.1 image analysis software (Perkin-Elmer, Boston, USA) running on an iMac computer (27 inch with OS Yosemite, v. 10.10).

### 2.9. Production and characterization of antibodies to ArSS2

To enable generation of rabbit antisera to ArSS2, synthetic ArSS2 (RAKNARCMADFWKGRGLVDC, with a disulfide bridge between the cysteines; custom synthesized by Peptide Protein Research Ltd, Fareham, UK) was conjugated to the carrier protein porcine thyroglobulin (Sigma-Aldrich, Gillingham, UK) using 5% glutaraldehyde (v/v) in phosphate buffer (PB, 0.1 M sodium phosphate dibasic and 0.1 M sodium phosphate monobasic, pH 7.2) as a coupling reagent. The coupling reaction products were dialysed in PBS at 4°C for approximately 48 h to remove low molecular mass components and then the purified conjugate was aliquoted and stored at −20°C. Rabbit immunization and serum collection was performed by Charles River Labs (Margate, UK). On day 0, pre-immune serum was collected and the first immunization was administered. Booster immunizations were administered on days 28, 42 and 56. Antiserum samples were collected on days 37 and 51 and a final bleed was collected on day 70. To assess production of antibodies during the immunization protocol and following collection of a terminal bleed, enzyme-linked immunosorbent assays (ELISA) were carried out to test antisera for the presence of antibodies to ArSS2. One hundred microlitres of a 1 µM solution of antigen peptide was added to each well of a PVC microtitre plate (Starlab, Milton Keynes, UK) overnight at 4°C. After washing with PBS, 200 µl 5% normal goat serum (Sigma-Aldrich, Gillingham, UK) was added to each well at room temperature for 2 h to block non-specific binding sites. Then after washing with PBST, varying dilutions of antiserum or pre-immune serum (10^−1^ to 10^−6^ diluted in 5% goat serum/PBS) were added to each well. After overnight incubation at 4°C, the antiserum solutions were washed away and each well was incubated with alkaline phosphatase (AP)-labelled goat anti-rabbit IgG secondary antibodies (Thermo Fisher Scientific, Oxford, UK; diluted 1 : 3000 in 5% goat serum/PBS) for 3 h at room temperature. Then after washing with PBST the *p*-nitrophenylphosphate alkaline phosphatase substrate (Vector Laboratories, Peterborough, UK) was added to each well for a 20 min incubation at room temperature. Lastly, absorbance at 415 nm was measured using a FLUOstar Omega plate reader (BMG LABTECH, Ortenberg, Germany) and then the mean absorbance values were calculated and plotted using Prism 6.

### 2.10. Localization of ArSS2 in *A. rubens* using immunohistochemistry

Small or medium-sized adult specimens of *A. rubens* were fixed in Bouin's solution (75% saturated picric acid in seawater, 25% formalin, 5% acetic acid) for at least 2 days at 4°C and then dissected to separate the arms and central disc. These body parts were then decalcified in 1% ascorbic acid/0.15 M sodium chloride solution at 4°C for at least one week. After dehydration through an ethanol series, each arm or central disc was embedded in paraffin and then 10 μm sections were cut using a microtome (RM 2145, Leica Microsystems [UK], Milton Keynes, UK) and mounted on chrome alum/gelatin-coated microscope slides. The slides were incubated with xylene (3 × 10 min) to remove the paraffin and then immersed in 100% ethanol twice. 0.3% hydrogen peroxide (VWR) in methanol was used to quench endogenous peroxidase activity for 30 min and then an ethanol series (90, 70, 50%) was used to rehydrate the tissue sections. Slides were washed in distilled water and blocked in 5% goat serum (Sigma-Aldrich, Gillingham, UK) made up in PBST for 2 h at room temperature. Finally, the slides were incubated at 4°C overnight with primary rabbit antiserum to ArSS2 (diluted 1 : 5000 in 5% goat serum/PBST). On the second day, slides were washed with PBST and incubated for 3 h with secondary antibodies (goat anti-rabbit horseradish peroxidase conjugated immunoglobulins (Jackson ImmunoResearch via Stratech Scientific, Newmarket, Suffolk, UK)) diluted 1 : 3000 in 2% goat serum/PBST. Immunostaining was produced using a staining solution comprising 0.05% diaminobenzidine (VWR Chemicals, Lutterworth, UK), 0.05% nickel chloride, 0.015% hydrogen peroxide diluted in PBS. Distilled water was used to wash slides when the staining was observed. Following dehydration through washes in autoclaved distilled water, ethanol (50, 70, 90, 100%) and xylene, slides were mounted with coverslips using DPX (Thermo Fisher Scientific, Oxford, UK) as a mounting medium. To assess the specificity of immunostaining, antiserum pre-absorption was performed by incubating the antiserum (diluted to 1 : 500 in PBS) with the ArSS2 antigen peptide (20 µM) on a rocking shaker at room temperature for 2 h. Then the pre-absorbed antiserum was diluted 10-fold to 1 : 5000 in 5% goat serum/PBST and tested on starfish sections, as described above. Images of stained sections were captured as described in §2.8.

### 2.11. Analysis of the *in vitro* pharmacological effects of ArSS2 on tube foot, apical muscle and cardiac stomach preparations from *A. rubens*

Synthetic ArSS2 (RAKNARCMADFWKGRGLVDC, with a disulfide bridge between the cysteine residues) was custom synthesized (Peptide Protein Research Ltd, Fareham, UK) to enable testing of its pharmacological effects on *in vitro* preparations of organs/muscles from *A. rubens*. ArSS2 was tested for its *in vitro* effects on the contractility of tube foot, apical muscle and cardiac stomach preparations from at least six different specimens of *A. rubens*. Individual tube foot preparations were dissected from starfish arms with their corresponding ampulla intact. Then cotton ligatures were tied around the ambulacral body wall and the tube foot disc and then the external epithelium of the tube foot was scraped off using a blunt scalpel blade to facilitate penetration of peptides when tested *in vitro*, as described previously [64,65]. Apical muscle preparations were dissected from the aboral body wall with approximately 1 cm strips of apical muscle tied at each end with cotton ligatures [64]. Cardiac stomach preparations were dissected as described previously [53,65] and then were tied at the aboral side of the cardiac stomach and around the oesophagus with cotton ligatures. The dissected preparations were tied to a fixed metal hook orally and to a high-grade isotonic transducer (MLT0015; ADInstruments Ltd; Oxford, UK) aborally, suspended in a 20 ml aerated organ bath containing artificial seawater at approximately 11°C. The transducer was connected *via* a bridge amplifier (FE221 Bridge Amp, ADInstruments Ltd, Oxford, UK) to data acquisition hardware (PowerLab 2/36, ADInstruments Ltd, Oxford, UK). Data collected from PowerLab were visualized as traces and analysed using LabChart (v. 8.0.7) software installed on a laptop computer (Lenovo E540, Windows 7 Professional). Preliminary tests revealed that ArSS2 caused relaxation of tube foot and cardiac stomach preparations but had no effect on the contractile state of apical muscle preparations. Therefore, the relaxing effects of ArSS2 on tube foot and cardiac stomach preparations were investigated in more detail.

To examine the effects of ArSS2 on tube foot and cardiac stomach preparations, 10^−6^ M acetylcholine (ACh) or artificial seawater (ASW) containing 30 mM added KCl was used to induce contraction of these preparations, respectively. Once a stable baseline contracted state was achieved, synthetic ArSS2 was added into the organ bath to sequentially achieve final concentrations in the range of 10^−10^ to 10^−6^ M. For these experiments, the contracting effect of 10^−6^ M ACh or ASW containing 30 mM added KCl was defined as 100% and the relaxing effects of ArSS2 were then calculated as a percentage reversal of the 100% contracted state.

To enable assessment of the magnitude of the relaxing effect of ArSS2 on the cardiac stomach, experiments were performed to compare the effect of ArSS2 with the effects of the neuropeptides SALMFamide-2 (S2; SGPYSFNSGLTF-NH_2_) and asterotocin (CLVQDCPEG-NH_2_ with a disulfide bridge between the cysteines), both of which cause relaxation of *A. rubens* cardiac stomach preparations *in vitro* [65–67]. Experiments were performed where the effects of ArSS2, S2 and asterotocin (at 10^−7^ and 10^−6^ M) on cardiac stomach preparations (*n* = 6) were compared. For these experiments, the effect of 10^−7^ or 10^−6^ M S2 was defined as 100% and the effect of asterotocin or ArSS2 was calculated as a % of the effect of S2. To determine if there were significant differences in magnitude of the relaxing effects of the three peptides on cardiac stomach preparations, data were analysed statistically using a two-tailed Student's *t*-test in Prism 6.

### 2.12. Analysis of the *in vivo* pharmacological of effects of ArSS2 on *A. rubens*

When starfish (*A. rubens*) feed, the cardiac stomach is relaxed and everted out of the mouth over the digestible soft tissues of prey (e.g. mussels) [68,69]. Previous studies have revealed that S2 and asterotocin induce relaxation of *A. rubens* cardiac stomach preparations *in vitro* and also induce cardiac stomach eversion when injected *in vivo* [66,67]. Therefore, because ArSS2 causes relaxation of cardiac stomach preparations *in vitro*, we investigated if ArSS2 also triggers cardiac stomach eversion when injected *in vivo*. Firstly, 15 medium-sized starfish were starved for one week to normalize their physiological status. A Hamilton 75N 50 µl syringe (Sigma-Aldrich; Gillingham, UK) was used to inject 10 µl 1 mM ArSS2, or 10 µl 1 mM asterotocin (positive control) or 10 µl water (negative control) into the perivisceral coelom of starfish. ArSS2 was tested on five individuals firstly and then the next day, asterotocin was tested on the same batch of five individuals. For another five individuals, asterotocin was tested first and then ArSS2 was tested on the following day. The remaining five individuals were injected with water as a control. Thus, both ArSS2 and asterotocin were tested on ten starfish. For each starfish, the test agent was injected via two sites in the dorsal body wall of the arms proximal to the junctions with the central disc region. Care was taken to inject test agents into the perivisceral coelom and not into the cardiac stomach. Starfish were placed individually in a transparent Petri dish containing ASW and their activity was video recorded for 10 min using a camera (Canon EOS 700D) positioned underneath them so that their oral surface could be viewed. Static images from video recordings were captured at 1 min intervals from the time of injection and if stomach eversion occurred the two-dimensional area of the everted cardiac stomach was measured using ImageJ software (https://imagej.nih.gov/ij/) and then normalized as a percentage of the area of the central disc region.

## 3. Results

### 3.1. Sequencing of cDNAs encoding the *A. rubens* neuropeptide precursors ArSSP1 and ArSSP2 and mass spectrometric characterization of neuropeptides (ArSS1 and ArSS2) derived from these precursors in extracts of *A. rubens* radial nerve cords

**Figure 1.**
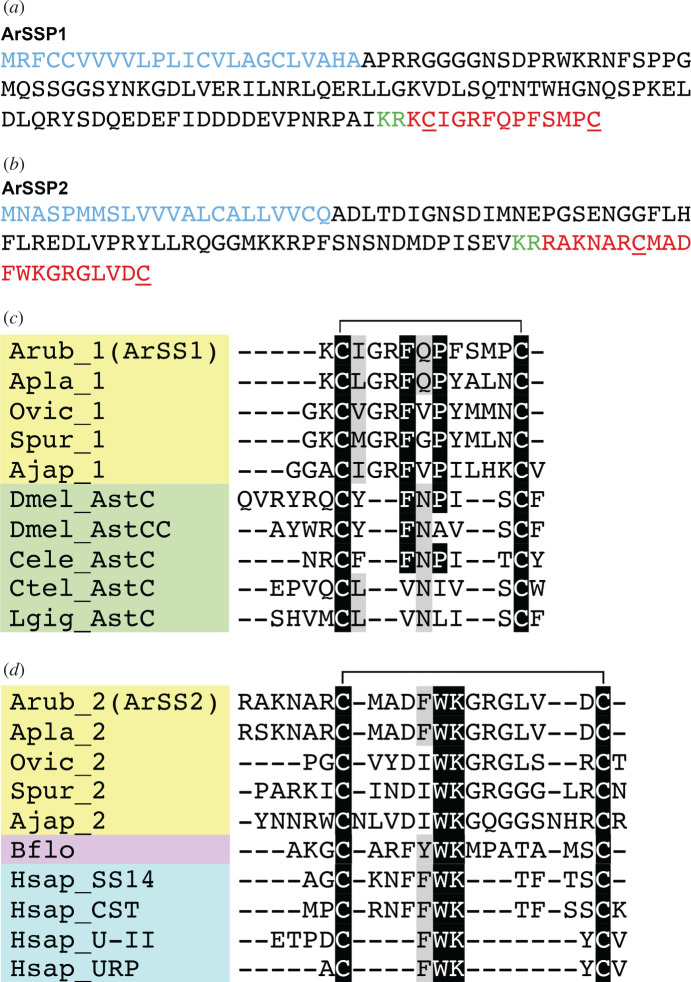
Sequences of the *A. rubens* neuropeptide precursors ArSSP1 and ArSSP2 and alignment of neuropeptides derived from these precursors (ArSS1 and ArSS2) with related peptides from other taxa. (*a*) Amino acid sequence of ArSSP1, with the predicted signal peptide shown in blue, a dibasic cleavage site shown in green and the neuropeptide ArSS1 shown in red. The underlined cysteine residues are predicted to form a disulfide bridge in the mature ArSS1 peptide. (*b*) Amino acid sequence of ArSSP2, with the predicted signal peptide shown in blue, a dibasic cleavage site shown in green and the neuropeptide ArSS2 shown in red. The underlined cysteine residues are predicted to form a disulfide bridge in the mature ArSS2 peptide. (*c*) Alignment of echinoderm SS1-type neuropeptides with protostome ASTC-type peptides. Note that in addition to conserved cysteine residues, there is a conserved FXP motif (where X is variable). A GR motif is, more specifically, a shared feature of the echinoderm peptides. (*d*) Alignment of echinoderm SS2-type neuropeptides with chordate SS-type peptides. Note that in addition to conserved cysteine residues, there is a conserved FWK/IWK motif. An aspartate (D) residue and two glycine (G) residues are, more specifically, shared features of the echinoderm peptides. In (*c*,*d*), the aligned amino acids are highlighted in black if the residue is present in at least 70% of the sequences or highlighted in grey if conservative amino acid substitutions are present in at least 70% of the sequences. The position of a disulfide bridge between the two conserved cysteine residues is shown above the alignments. Species and peptide names are highlighted in taxon-specific colours: yellow (Echinodermata), green (Protostomes), pink (Cephalochordata), blue (Vertebrata). Species name abbreviations are as follows: Ajap, *Apostichopus japonicus*; Apla, *Acanthaster planci*; Arub, *Asterias rubens*; Bflo, *Branchiostoma floridae*; Cele, *Caenorhabditis elegans*; Ctel, *Capitella teleta*; Dmel, *Drosophila melanogaster*, Hsap, *Homo sapiens*, Lgig, *Lottia gigantea*; Ovic, *Ophionotus victoriae*; Spur, *Strongylocentrotus purpuratus*.

Cloning and sequencing of a cDNA encoding ArSSP1 (electronic supplementary material, figure S1) confirmed a transcript sequence (contig 1107850) from *A. rubens* radial nerve cord transcriptome data, which was previously referred to as ArSSP (GenBank: KT601708) [48]. ArSSP1 is a 132-residue protein comprising a predicted 24-residue N-terminal signal peptide and the C-terminally located predicted SS-like peptide ArSS1 (figure 1*a*). Analysis of *A. rubens* radial nerve cord extracts using mass spectrometry enabled determination of the mature structure of ArSS1, revealing that it is a 13-residue peptide (KCIGRFQPFSMPC) that was identified as a 2+ charged precursor mass 814.38 *m/z*. This peptide was identified in samples of extracts that were subjected to reduction and alkylation and accordingly, the two cysteine residues were modified by carbamidomethylation, as seen in the MS/MS mass spectrum by higher-energy collisional dissociation (HCD) fragmentation (electronic supplementary material, figure S3a). It is not possible to fragment cyclic peptides that have a disulfide bridge using the standard MS/MS HCD fragmentation and accordingly in samples of extracts where reduction and alkylation was not performed, the ArSS1 peptide was not identified. Collectively, these findings demonstrate that the peptide ArSS1 has the amino acid sequence KCIGRFQPFSMPC with a disulfide bridge between the two cysteine residues and is present in *A. rubens* radial nerve cord extracts.

Analysis of transcriptome sequence data from the brittle star *O. victoriae* has revealed the presence of two transcripts encoding SS-like neuropeptides [49], one of which is closely related to ArSSP1. Submission of the second *O. victoriae* precursor sequence as a query for BLAST analysis of *A. rubens* radial nerve cord transcriptome sequence data revealed the presence of a contig (contig 1111056) encoding a closely related precursor, which we have named ArSSP2 (GenBank: MN257487). Cloning and sequencing of a cDNA encoding ArSSP2 confirmed the contig sequence (electronic supplementary material, figure S2), revealing that it is a 102-residue protein, comprising a predicted 22-residue N-terminal signal peptide and the predicted C-terminally located SS-like peptide ArSS2 (RAKNARCMADFWKGRGLVDC) (figure 1*b*).

Analysis of *A. rubens* radial nerve cord extracts using mass spectrometry enabled determination of the mature structure of ArSS2, revealing that it is a 20-residue peptide (RAKNARCMADFWKGRGLVDC) that was identified as a 4+ charged precursor mass 603.55 *m/z*. This peptide was identified in samples of extracts that were subjected to reduction and alkylation and accordingly, the two cysteine residues were modified by carbamidomethylation. However, the MS/MS HCD fragmentation for this peptide was poor (electronic supplementary material, figure S3b) and it was not possible to confirm the full amino acid sequence. As expected, this peptide was not identified in the samples that were not subject to reduction and alkylation, providing supporting evidence of the presence of a disulfide bridge as discussed above for ArSS1.

### 3.2. Sequence alignment of ArSS1 and ArSS2 with other SS/ASTC-type peptides

Comparison of the sequences of ArSS1 and ArSS2 with SS/ASTC-type neuropeptides from other species revealed several conserved features. All of the peptides contain a pair of cysteine residues, which are predicted or known to form an intramolecular disulfide bridge that confers a cyclic structure on the mature peptides (figure 1*c,d*). Furthermore, ArSS1 and other SS1-type peptides from echinoderms have a conserved FXP motif (where X is variable) in common with ASTC-type peptides from *D. melanogaster* and *Caenorhabditis elegans* (figure 1*c*), while ArSS2 and SS2-type peptides from other echinoderms have a conserved FWK/IWK motif in common with SS-type peptides from chordates (figure 1*d*). Echinoderm-specific conservation of other residues in SS1-type and SS2-type peptides can also been seen in figure 1*c*,*d*, respectively. An alignment that combines the neuropeptide sequences shown separately in figure 1*c*,*d* is presented in electronic supplementary material, figure S4.

### 3.3. Comparison of the structure of genes encoding SS1- and SS2-type neuropeptides in echinoderms and genes encoding SS/ASTC/UII-type neuropeptides in other taxa

**Figure 2.**
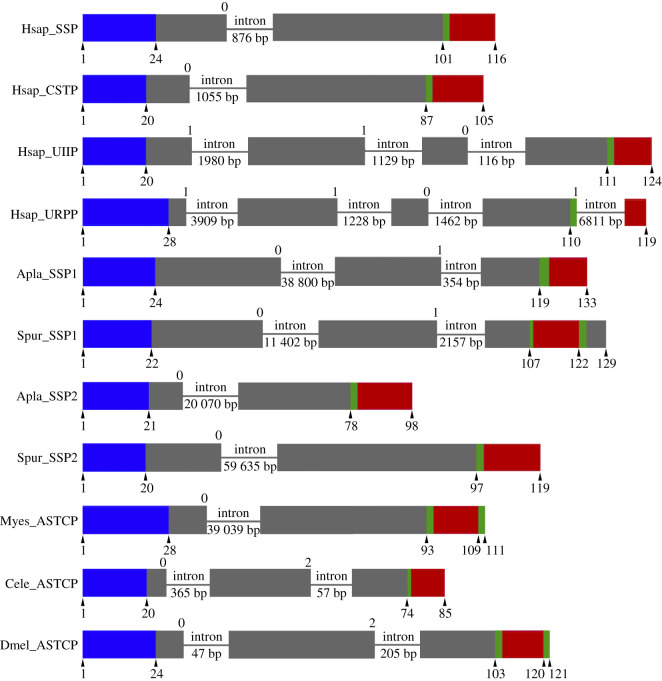
Comparison of the exon/intron structure of genes encoding somatostatin/allatostatin-C-related precursors in echinoderms and other taxa. The figure shows representations of the gene structures. The protein-coding exons are shown as rectangles and introns are shown as lines with intron length stated underneath. The N-terminal signal peptide, the neuropeptide and monobasic or dibasic cleavage sites are shown in blue, red and green, respectively, with amino acid residue positions shown underneath. Other regions of the precursor protein are shown in grey. The numbers above each precursor diagram show the phase of introns with respect to the reading frame, with 0 representing an intron located between two consecutive codons, 1 representing an intron between the first and second base of a codon and 2 representing an intron between the second and third base of a codon. Note that a shared characteristic of the echinoderm SSP1-type and SSP2-type genes and SS/CST/ASTC-type genes in other taxa is the presence of a phase 0 intron interrupting the coding region for the N-terminal part of the encoded proteins; this distinguishes these genes from UII/URP-type genes that have a phase 1 intron in an equivalent position. Species names are as follows: Apla, *Acanthaster planci*; Cele, *Caenorhabditis elegans*; Dmel, *Drosophila melanogaster*; Hsap, *Homo sapiens*; Myes, *Mizuhopecten yessoensis*; Spur, *Strongylocentrotus purpuratus*. The neuropeptide precursor names are as follows: SS, somatostatin; CST, cortistatin; UII, urotensin II; URP, urotensin II-related peptide; ASTC, allatostatin-C. Accession numbers for the sequences of the precursors shown in this figure are listed in electronic supplementary material, table S3.

To further investigate relationships between echinoderm SS-like peptides and SS/ASTC-type peptides in other taxa, we compared the positions of introns in the protein-coding regions of genes encoding these peptides (figure 2). The *H. sapiens* SS and cortistatin (CST) genes both have a phase 0 intron interrupting exons encoding the N-terminal part of the precursor proteins. This contrasts with the *H. sapiens* urotensin II (UII) and Urotensin II-related peptide (URP) genes, which both have a phase 1 intron interrupting exons encoding the N-terminal part of the precursor proteins as well as two or three introns interrupting exons encoding the C-terminal part of the precursor proteins. Genes encoding ASTC-type neuropeptides in protostome invertebrates (the arthropod *D. melanogaster*, the nematode *C. elegans*, the mollusc *Mizuhopecten yessoensis*) are similar to human SS/CST genes in having a phase 0 intron interrupting exons encoding the N-terminal part of the precursor proteins. However, the *D. melanogaster* and *C. elegans* ASTC-type genes also have a phase 2 intron interrupting exons encoding the C-terminal part of the precursor proteins. Thus, the presence of a phase 0 intron interrupting exons encoding the N-terminal part of the precursor protein appears to be a conserved characteristic of SS/ASTC-type precursor genes. It is noteworthy, therefore, that both the SS1-type and SS2-type precursor genes in the starfish *A. planci* and the sea urchin *S. purpuratus* have a phase 0 intron interrupting exons encoding the N-terminal part of the precursor protein. However, the SS1-type neuropeptide genes also have a phase 1 intron that interrupts exons encoding the C-terminal part of the precursor proteins. Collectively, these findings provide evidence that the echinoderm SS1- and SS2-type precursor genes are homologues of SS/ASTC-type genes in other taxa.

### 3.4. Identification of three transcripts encoding orthologues of SS/ASTC-type receptors in *A. rubens*

**Figure 3.**
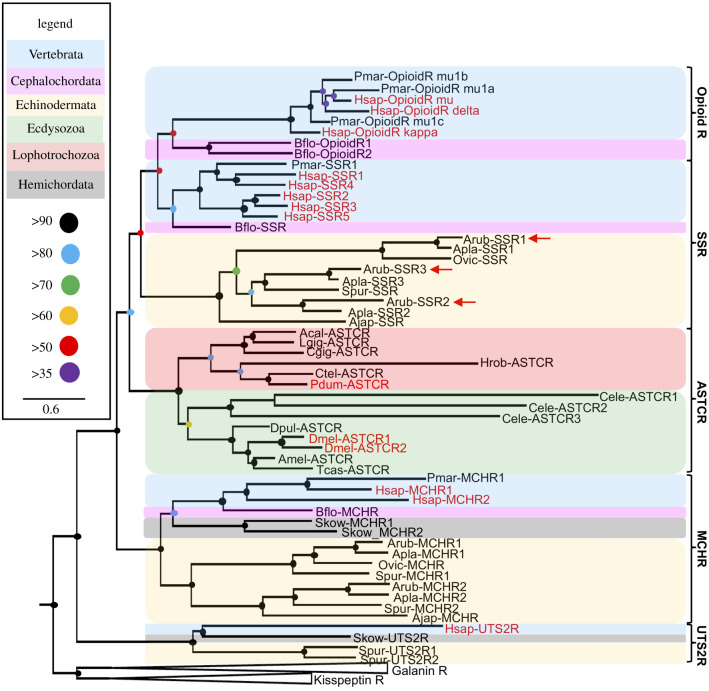
Phylogenetic analysis identifies three *A. rubens* receptor proteins as somatostatin/allatostatin-C-type receptors. The tree generated using the maximum-likelihood method (1000 bootstrap replicates, LG + F + I + G4 substitution model) comprises three distinct receptor clades—SS/ASTC/opioid-type receptors, MCH-type receptors and urotensin II-type receptors. Galanin-type receptors and kisspeptin-type receptors were included here as outgroups to root the tree. The three *A. rubens* receptor proteins characterized in this study (labelled with red arrows) are positioned in the clade containing SS/opioid/ASTC-type receptors, demonstrating that they can be classified as SS/ASTC-type receptors. The round dots represent bootstrap support and the different coloured backgrounds highlight different taxonomic groups (see legend). The scale bar represents the average residue substitution per site. Receptor names shown in red indicate that cognate ligands for these receptors have been identified experimentally. Species names are as follows: Acal, *Aplysia californica*; Ajap, *Apostichopus japonicus*; Amel, *Apis mellifera*; Apla, *Acanthaster planci*; Arub, *Asterias rubens*; Bflo, *Branchiostoma floridae*; Cele, *Caenorhabditis elegans*; Cgig, *Crassostrea gigas*; Ctel, *Capitella teleta*; Dmel, *Drosophila melanogaster*; Dpul, *Daphnia pulex*; Hrob, *Helobdella robusta*; Hsap, *Homo sapiens*; Lgig, *Lottia gigantea*; Ovic, *Ophionotus victoriae*; Pdum, *Platynereis dumerilii*; Pmar, *Petromyzon marinus*; Skow, *Saccoglossus kowalevskii*; Spur, *Strongylocentrotus purpuratus*; Tcas, *Tribolium castaneum*. Accession numbers of receptor sequences and associated references used to generate this figure are listed in electronic supplementary material, table S4.

To identify candidate receptors for ArSS1 and/or ArSS2 in *A. rubens*, SS/ASTC-type receptor protein sequences were submitted as queries for BLAST-based analysis of *A. rubens* neural transcriptome sequence data. Contigs encoding five proteins (contigs 1120385, 1108044, 1107700, 1119135 and 1121233) were identified as the top hits and homologues of these proteins were also identified in other echinoderms, including the starfish *A. planci*, the brittle star *O. victoriae,* the sea urchin *S. purpuratus* and the sea cucumber *A. japonicus*. To determine the relationship of these echinoderm receptors with other bilaterian neuropeptide receptors, phylogenetic analysis was carried out using the maximum-likelihood method. In addition to bilaterian SS-type receptors and protostome ASTC-type receptors, the following receptors that are closely related to SS/ASTC-type receptors were also included in this analysis—opioid-type receptors, MCH-type receptors and urotensin II-type receptors. Galanin-type receptors and kisspeptin-type receptors were included as outgroups. This revealed that three of the five candidate receptors identified in *A. rubens* (contigs 1120385, 1108044, 1107700) were positioned in a clade comprising chordate SS-type receptors, chordate opioid-type receptors and protostome ASTC-type receptors. However, the precise position of the echinoderm receptors within this clade was dependent on the extent of sequence trimming, with more trimming placing the echinoderm receptors in a branch comprising chordate SS-type and chordate opioid-type receptors (figure 3) and with less trimming placing the echinoderm receptors in a branch comprising protostome ASTC-type receptors (electronic supplementary material, figure S5). Therefore, these receptors were designated as SS/ASTC-type receptors, but for simplicity were named ArSSR1 (contig 1120385), ArSSR2 (contig 1108044) and ArSSR3 (contig 1107700) (figure 3). The other two *A. rubens* receptors (contigs 1119135 and 1121233) were positioned in a clade comprising MCH-type receptors, and therefore these were named ArMCHR1 and ArMCHR2, respectively (figure 3).

### 3.5. Pharmacological characterization of the *A.rubens* SS/ASTC-type receptors ArSSR1, ArSSR2 and ArSSR3

**Figure 4.**
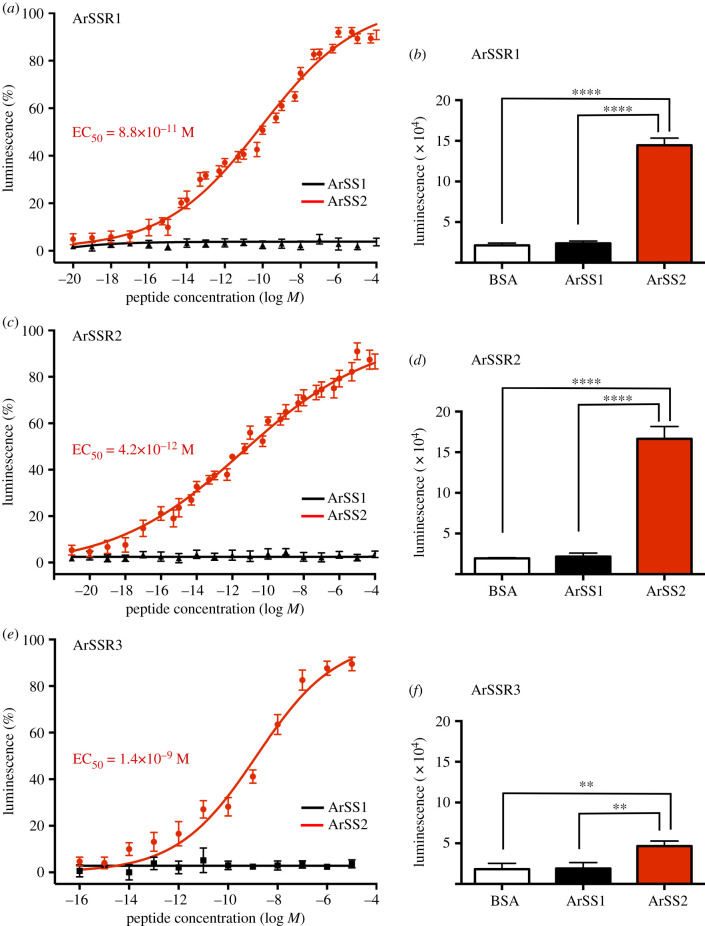
ArSS2, but not ArSS1, acts as a ligand for three *A. rubens* SS/ASTC-type receptors, ArSSR1, ArSSR2 and ArSSR3. The graphs show that ArSS2 causes dose-dependent activation of ArSSR1 (*a*), ArSSR2 (*c*) and ArSSR3 (*e*) expressed with Gα16 in CHO-K1 cells stably expressing a calcium-sensitive bioluminescent GFP-aequorin fusion protein G5A. Luminescence is expressed as a percentage of the maximal response observed in each experiment (*a*,*c*,*e*) and the ArSS2 mean (±s.e.m.) EC_50_ values for each receptor are shown in red lettering. ArSS1 has no effect when tested over the same concentration range as ArSS2, demonstrating the specificity of the activation of ArSSR1–3 by ArSS2 (*a*,*c*,*e*). Each point represents mean values (±s.e.m.) from at least three independent experiments, with three repeats per experiment. (*b*,*d*,*f*) Comparison of the total bioluminescence responses of ArSSR1 (*b*), ArSSR2 (*d*) and ArSSR3 (*f*) expressing cells when exposed to ArSS2 and ArSS1 at a concentration of 10^−5^ M for 30 s. Cells exposed to BSA media were used as a control group. The mean luminescence responses to 10^−5^ M ArSS2 are 144 620 (*b*), 166 303 (*d*) and 46 575 (*f*), respectively, showing that ArSS2 has higher efficacy as ligand for ArSSR1 and ArSSR2 than for ArSSR3. Analysis of the data in (*b*,*d*,*f*) by one-way ANOVA with Bonferroni's multiple comparisons *post hoc* test revealed that luminescence in cells exposed to 10^−5^ M ArSS2 is significantly higher (****p < 0.0001, **p < 0.01) than in cells exposed to 10^−5^ M ArSS1 or BSA media (control). Luminescence is not significantly different between receptor-expressing cells exposed to 10^−5^ M ArSS1 and receptor-expressing cells exposed to BSA media.

Having identified ArSSR1, ArSSR2 and ArSSR3 as candidate receptors for ArSS1 and/or ArSS2, cDNAs encoding these proteins were cloned and sequenced so that ArSS1 and ArSS2 could be tested as ligands for these receptors. Cloning and sequencing of cDNAs encoding ArSSR1, ArSSR2 and ArSSR3 confirmed sequences obtained from *A. rubens* radial nerve cord transcriptome sequence data (electronic supplementary material, figures S6–S8) and these sequences have been deposited in GenBank under accession numbers MN251831, MN251832 and MN251833, respectively. ArSSR1, ArSSR2 and ArSSR3 comprise 346, 418 and 341 amino acid residues, respectively, and sequence analysis using Protter indicated the presence of seven predicted transmembrane domains, as expected for G-protein-coupled receptors (electronic supplementary material, figures S9–S11). The cloned receptor cDNAs were co-transfected with Gα16 into CHO-cells stably expressing GFP-aequorin so that synthetic ArSS1 and ArSS2 could be tested as candidate ligands for these receptors at concentrations ranging from 10^−21^ to 10^−4^ M. ArSS1 did not trigger luminescence in cells expressing any of the three receptors, even at the highest concentration tested. However, ArSS2 caused dose-dependent luminescence in cells expressing ArSSR1, ArSSR2 and ArSSR3, with half-maximal response concentrations (EC_50_) of 8.8 × 10^−11^ M, 4.2 × 10^−12^ M and 1.4 × 10^−9^ M, respectively (figure 4*a*,*c*,*e*). At a concentration of 10^−5^ M, ArSS2 triggered mean total luminescence responses in CHO-cells expressing ArSSR1, ArSSR2 or ArSSR3 that were, respectively, 144 620 (figure 4*b*), 166 303 (figure 4*d*) and 46 575 (figure 4*f*) times the background luminescence detected with the assay media used to dissolve the peptides or with ArSS1. Thus, the efficacy of ArSS2 as a ligand was higher for ArSSR1 and ArSSR2 than for ArSSR3. Furthermore, statistical analysis of the data from these experiments showed that for all three receptors, the luminescence in cells exposed to ArSS2 was significantly higher than the luminescence in cells exposed to ArSS1 or BSA media (control). Neither ArSS1 nor ArSS2 elicited responses when tested on CHO-K1 cells transfected with an empty vector (electronic supplementary material, figure S12). Thus, these experiments demonstrated that ArSS2, but not ArSS1, acts as a ligand for the *A. rubens* receptors ArSSR1, ArSSR2 and ArSSR3. Therefore, we proceeded to investigate the physiological roles of ArSS2 in *A. rubens* by analysing its expression and pharmacological actions, as described below.

### 3.6. Localization of ArSSP2 transcripts in *A. rubens* using mRNA *in situ* hybridization

**Figure 5.**
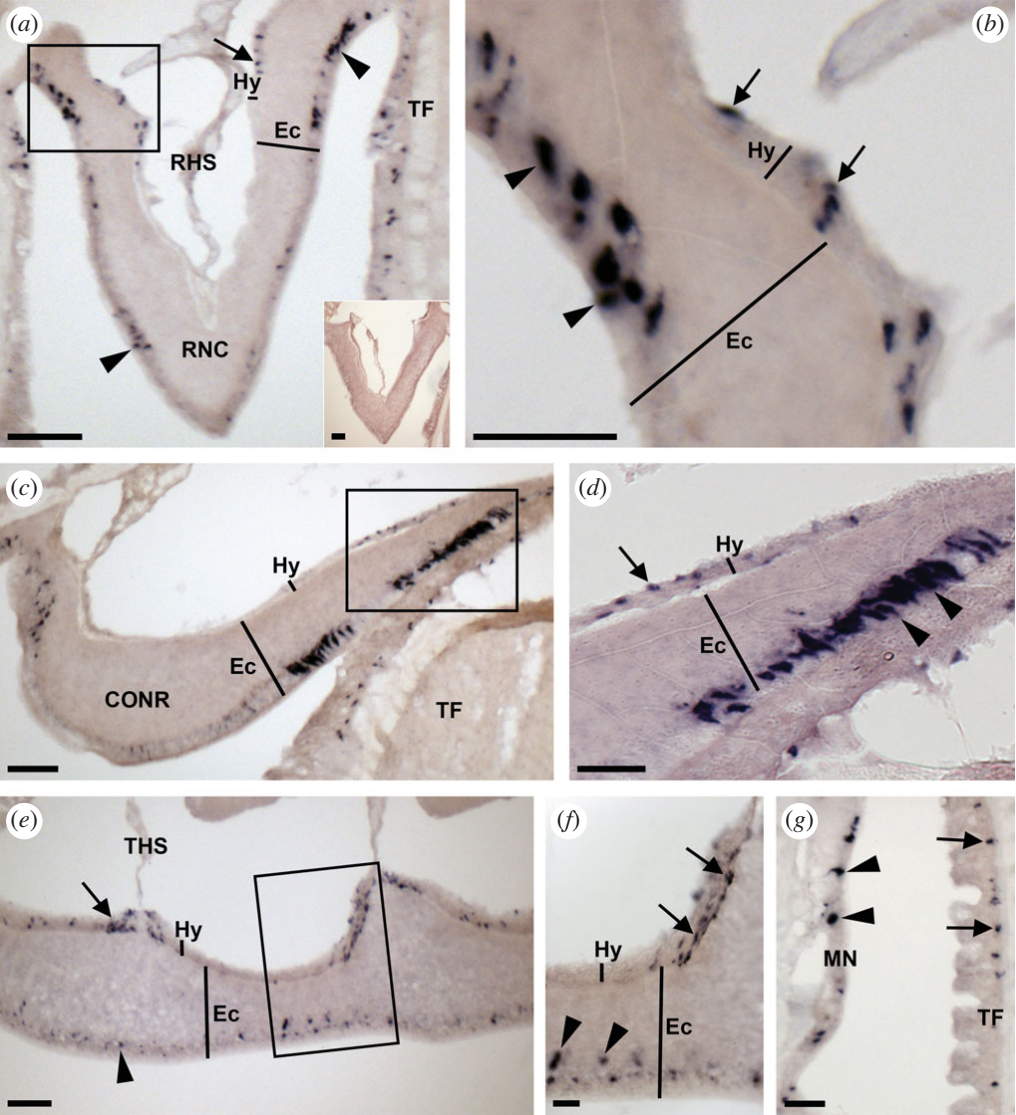
Localization of ArSSP2 mRNA in the nervous system of *A. rubens* using *in situ* hybridization. (*a*) Transverse section of a radial nerve cord incubated with antisense probes showing stained cells (ArSSP2-expressing cells) in both the hyponeural (arrow) and ectoneural (arrowheads) regions. The inset shows the absence of staining in a transverse section of radial nerve cord incubated with sense probes, demonstrating that staining observed with antisense probes can be attributed to the detection of ArSSP2 transcripts. (*b*) Higher magnification image of the boxed region in (*a*), showing stained cells in the hyponeural region (arrows) and in the subcuticular epithelial layer of the ectoneural region (arrowheads). (*c*) Transverse section of the central disc region showing stained cells in the hyponeural and ectoneural regions of the circumoral nerve ring; stained cells can also be seen in an adjacent peri-oral tube foot. (*d*) Higher magnification image of the boxed region in (*c*), showing stained cells in the hyponeural region (arrow) and the subcuticular epithelial layer of the ectoneural region (arrowheads). (*e*) Longitudinal parasagittal section of a radial nerve cord showing stained cells in both the hyponeural (arrow) and ectoneural (arrowhead) regions. Note that in the hyponeural region, the stained cells are clustered on either side of the transverse hemal strand. (*f*) Higher magnification image of the boxed region in (*e*), showing stained cells in the hyponeural region (arrows) and the subcuticular epithelial layer of the ectoneural region (arrowheads). (*g*) Stained cells in the body wall external epithelium proximal to the marginal nerve and in an adjacent tube foot (see also figure 7). CONR, circumoral nerve ring; Ec, ectoneural region; Hy, hyponeural region; MN, marginal nerve; RHS, radial hemal strand; RNC, radial nerve cord; TF, tube foot; THS, transverse hemal strand. Scale bars: 18 µm in (*f*); 30 µm in (*b*,*d*,*g*); 60 µm in (*a*), (*a*) inset, (*c*,*e*).

**Figure 6.**
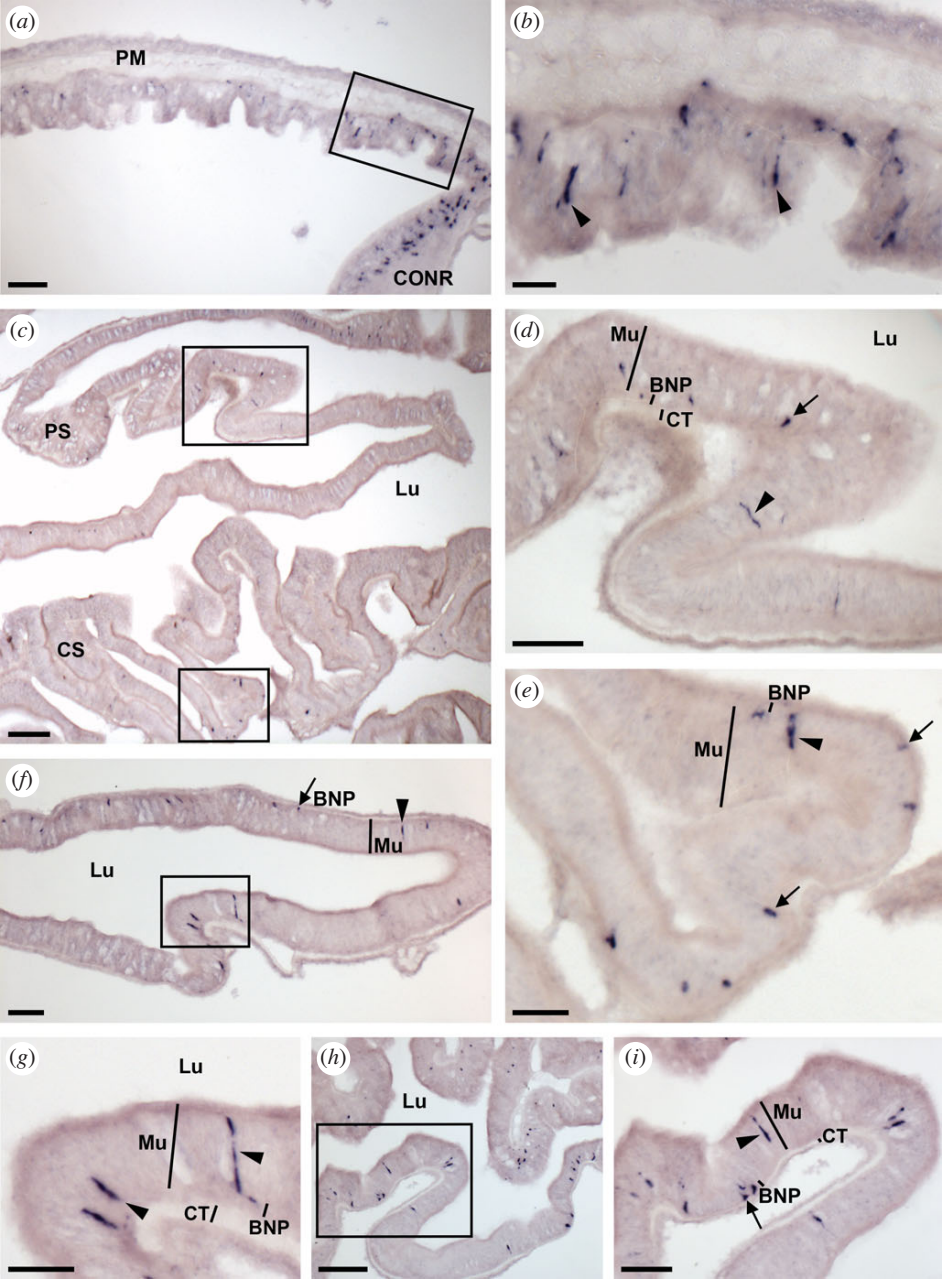
Localization of ArSSP2 mRNA in the digestive system of *A. rubens* using *in situ* hybridization. (*a*) Transverse section through the central disc region showing stained cells in the peristomial membrane and in the circumoral nerve ring that surrounds the peristomial membrane. (*b*) Higher magnification image of the boxed region shown in (*a*), showing stained cells (arrowheads) in the external epithelium of the peristomial membrane. (*c*) Transverse section through the central disc region showing sparsely distributed stained cells in the cardiac stomach and pyloric stomach. (*d*) Higher magnification image of the boxed region of the pyloric stomach in (*c*), showing elongate shaped stained cells (arrowhead) in the mucosal layer and roundish shaped stained cells (arrow) close to the position of basi-epithelial nerve plexus. (*e*) Higher magnification image of the boxed region of the cardiac stomach in (*c*), showing elongate shaped stained cells (arrowhead) in the mucosal layer and roundish shaped stained cells (arrows) close to the position of the basi-epithelial nerve plexus. (*f*) Transverse section of a pyloric duct showing elongate shaped stained cells (arrowhead) and roundish shaped stained cells (arrow) located on both the aboral (upper) and oral (lower) sides. (*g*) Higher magnification image of the boxed region of (*f*) showing elongate shaped stained cells (arrowheads) in the mucosal layer. (*h*) Transverse section of an arm showing stained cells in a pyloric caecum. (*i*) Higher magnification image of the boxed region in (*h*), showing elongate shaped stained cells (arrowhead) in the mucosal layer and roundish shaped stained cells (arrow) close to the position of basi-epithelial nerve plexus. BNP, basi-epithelial nerve plexus; CONR, circumoral nerve ring; CS, cardiac stomach; Lu, lumen; Mu, mucosal layer; PC, pyloric caecum; PM, peristomial membrane; PS, pyloric stomach. Scale bars: 18 µm in (*b*); 30 µm in (*a*,*e*); 36 µm in (*g*); 60 µm in (*d*,*f*,*i*); 120 µm in (*c*,*h*).

**Figure 7.**
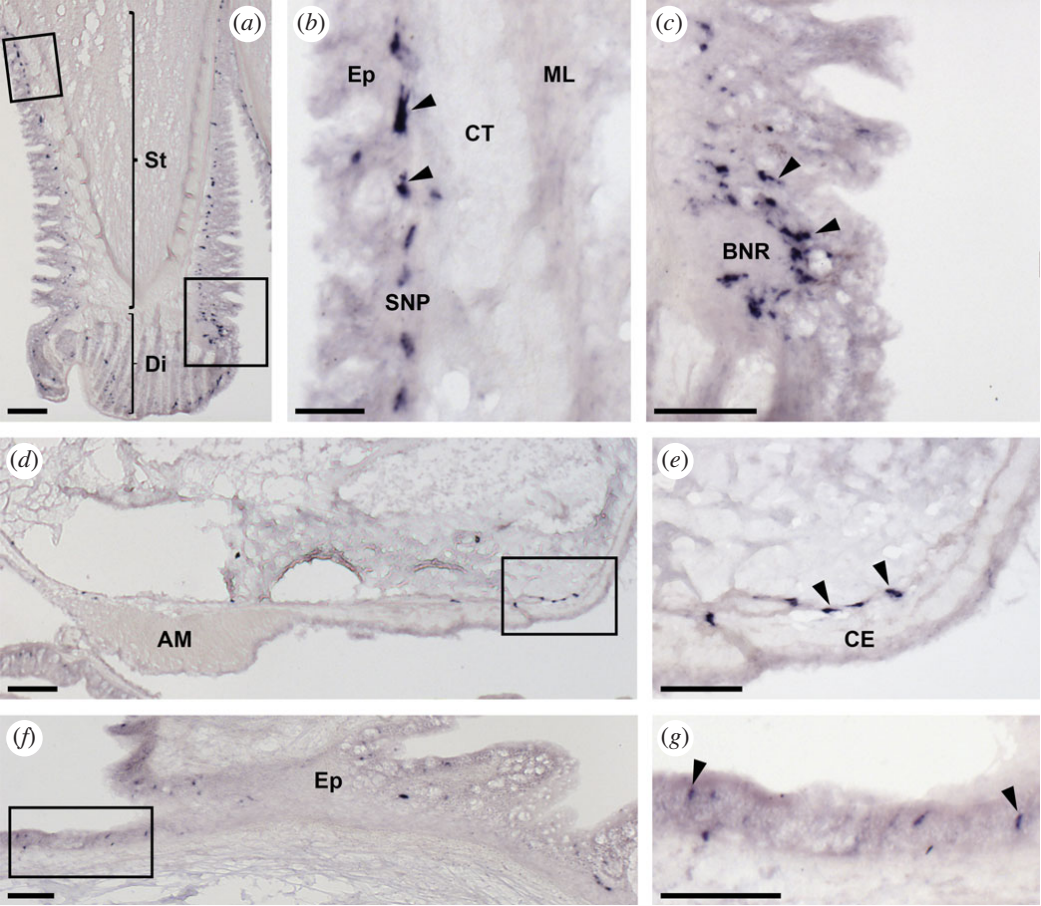
Localization of ArSSP2 mRNA in the tube feet and body wall of *A. rubens* using *in situ* hybridization. (*a*) Longitudinal section of a tube foot showing staining in both the stem and disc regions. (*b*) Higher magnification image of the stem region of (*a*), showing stained cells (arrowheads) in a subepithelial position. (*c*) Higher magnification image of the boxed region of (*a*) at the junction between the stem and the disc, showing a cluster of stained cells around the basal nerve ring. (*d*) Transverse section of the aboral body wall of an arm showing stained cells in the circular muscle layer proximal to the apical muscle. (*e*) Higher magnification image of the boxed region of (*d*), showing stained cells (arrowheads) in the circular muscle layer, which is located beneath the coelomic epithelium. (*f*) Transverse section of an arm showing staining in the external epithelium of the body wall. (*g*) Higher magnification image of the boxed region of (*f*), showing stained cells (arrowheads) in the external epithelium of the body wall. AM, apical muscle; BNR, basal nerve ring; CE, coelomic epithelium; CT, collagenous tissue; Di, disc; Ep, epithelium; ML, muscle layer; SNP, subepithelial nerve plexus; St, stem. Scale bars: 32 µm in (*b*); 60 µm in (*c*,*e*,*f*,*g*); 120 µm in (*a*,*d*).

Analysis of the expression of ArSSP2 transcripts in *A. rubens* using mRNA *in situ* hybridization revealed that ArSSP2 is widely expressed, with ArSSP2-expressing cells detected in the central nervous system (figure 5), digestive system (figure 6), tube feet and body wall (figure 7).

#### 3.6.1. Central nervous system

The central nervous system of *A. rubens* comprises radial nerve cords that are located on the underside of the five arms and a circumoral nerve ring in the central disc region. The radial nerve cords and circumoral nerve ring comprise two distinct regions—the ectoneural region that is thought to largely contain sensory neurons and interneurons, and the hyponeural region that contains only motor neurons. Marginal nerves run parallel with the radial nerve cords, lateral to the outer row of tube feet on each side of the arm [70–72].

Transverse sections of radial nerve cords revealed that ArSSP2 is expressed in both the ectoneural and hyponeural regions. In the ectoneural region, there are stained cells scattered throughout the subcuticular epithelial layer, but with bilaterally symmetrical clusters of strongly stained cells being most prominent close to the junction with adjacent tube feet (figure 5*a*,*b*). In the hyponeural region, a few stained cells can be observed in transverse sections (figure 5*a*,*b*). The absence of stained cells in sections incubated with sense probes confirmed the specificity of staining observed with antisense probes (figure 5*a* inset). Anatomically, the circumoral nerve ring corresponds to one half of the V-shaped radial nerve cord and the pattern of ArSSP2 transcript expression in the circumoral nerve ring was generally consistent with that in the radial nerve cords. In the ectoneural region of the circumoral nerve ring, there are two distinct bands of strongly stained cells located close to the junction with the neighbouring tube foot and another band of stained cells close to the junction with the peristomial membrane (figure 5*c*,*d*). In the hyponeural region of the circumoral nerve ring, only a few stained cells can be seen (figure 5*c*,*d*). Longitudinal sections of radial nerve cords revealed stained cells are distributed along the length of ectoneural subcuticular epithelium, whereas in the hyponeural region, there is segmental clustering of stained cells proximal to the transverse hemal strand (figure 5*e*,*f*). ArSSP2-expressing cells are also present in the epithelium overlying the marginal nerves (figure 5*g*).

#### 3.6.2. Digestive system

In *A. rubens* and other starfish, the mouth is located on the underside of the central disc and is surrounded by a contractile peristomial membrane, which is continuous with a short tubular oesophagus. Aboral to the oesophagus is the cardiac stomach, which is everted through the mouth during feeding. Aboral to the cardiac stomach is the smaller pyloric stomach, which is linked via a short rectum to the anus located on the aboral surface of the central disc and linked by pyloric ducts to paired pyloric caeca located in each arm [68,73]. ArSSP2 expression was revealed in several regions of the digestive system. In the peristomial membrane, stained cells were observed in the external epithelium in continuity with the ectoneural region of the circumoral nerve ring (figure 6*a*,*b*). In the cardiac stomach and pyloric stomach, sparse populations of stained cells were observed (figure 6*c*), with elongate shaped cells located in the mucosal layer and roundish shaped cells located close to the position of the basi-epithelial nerve plexus (figure 6*d*,*e*). Stained cells were also detected in the pyloric duct and pyloric caeca, located in the mucosal layer and basi-epithelial nerve plexus on both the oral and aboral sides of these parts of the digestive system (figure 6*f–i*).

#### 3.6.3. Body wall and tube feet

The body wall of *A. rubens* comprises an outer epithelium layer, a dermal layer and a coelomic epithelium layer. The dermal layer comprises calcite ossicles that are linked by muscles within collagenous tissue. Circularly and longitudinally orientated muscle layers are beneath the coelomic epithelium layer. The longitudinally orientated muscle layer is thickened in the midline of the aboral body wall of each arm to form the apical muscle. The external aboral surface of the body wall has many appendages, including pedicellariae, spines and papulae. The oral surface of the body wall comprises ambulacral ossicles, adambulacral ossicles and four rows of tube feet that are linked to ampullae located internally [74]. Stained cells expressing ArSSP2 were revealed in tube feet in a subepithelial position along the length of the stem (figure 7*a*,*b*), in close association with the basal nerve ring (figure 7*c*) and in the disc region (figure 7*a*). Stained cells expressing ArSSP2 were also revealed in the circular muscle layer beneath the coelomic epithelium (figure 7*d*,*e*) and in the external epithelium of the body wall (figure 7*f*,*g*).

### 3.7. Localization of ArSS2 expression in *A. rubens* using immunohistochemistry

Enzyme-linked immunosorbent assays (ELISA) were used to test for the presence of antibodies to ArSS2 in serum from a rabbit that had been immunized with a conjugate of thyroglobulin and ArSS2. Antibodies to ArSS2 were not detected in pre-immune serum, as expected. Analysis of serum from the final bleed revealed that antibodies to ArSS2 could be detected with serum dilutions as low as 10^−4^ (electronic supplementary material, figure S13). The rabbit antiserum to ArSS2 has been assigned the RRID: AB_2847910.

**Figure 8.**
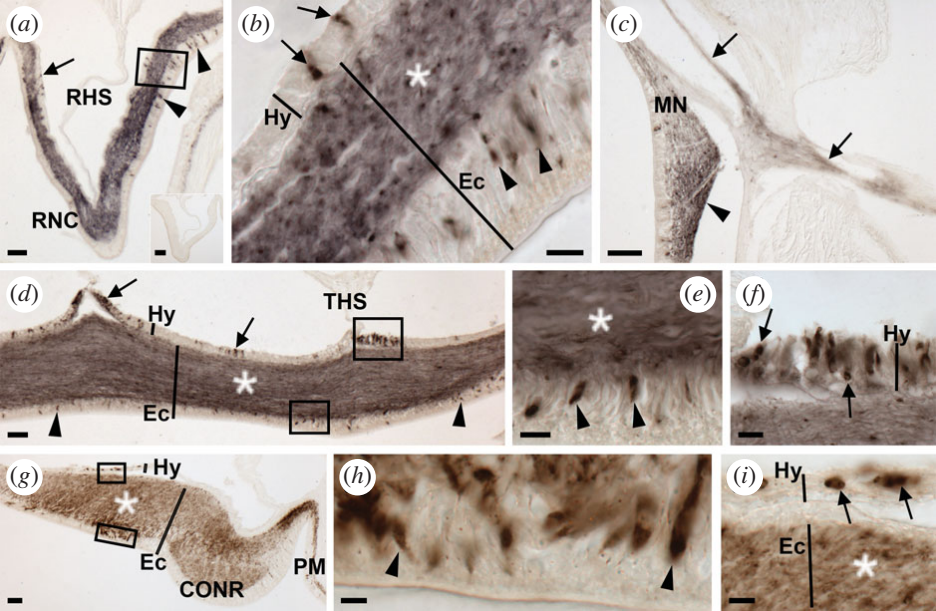
Localization of ArSS2 in the nervous system of *A. rubens* using immunohistochemistry. (*a*) Transverse section of the radial nerve cord showing ArSS2-immunoreactivity (immunostaining) in the hyponeural (arrows) and ectoneural (arrowheads) regions. In the ectoneural region, immunostained cells are located in the subcuticular epithelial layer, largely concentrated laterally, and immunostained fibres are present in the neuropile. The inset shows an absence of immunostaining in sections of radial nerve cord incubated with ArSS2 antiserum pre-absorbed with the ArSS2 peptide antigen, demonstrating the specificity of immunostaining observed in sections incubated with the ArSS2 antiserum. (*b*) Higher magnification image of the boxed region of (*a*), showing immunostained bipolar shaped cells in the subcuticular epithelium of the ectoneural region (arrowheads), immunostained roundish shaped monopolar cells in the hyponeural region (arrows) and immunostained fibres in the neuropile (asterisk). (*c*) Immunostaining in the marginal nerve (arrowhead) and immunostained fibres in the lateral motor nerve (arrows). (*d*) Longitudinal parasagittal section of a radial nerve cord showing immunostained cells in the hyponeural region (arrows) and ectoneural region (arrowheads). In the hyponeural region, immunostained cells are largely clustered near to the transverse hemal strand, whereas in the ectoneural region, immunostained cells are distributed evenly along the length of the radial nerve cord. Immunostained fibres are present throughout the ectoneural neuropile (asterisk). (*e*) Higher magnification image of the boxed ectoneural region of (*d*), showing immunostained bipolar shaped cells in the subcuticular epithelium (arrowheads) and immunostained fibres in the ectoneural neuropile (asterisk). (*f*) Higher magnification image of the boxed hyponeural region of panel (*d*), showing immunostained roundish monopolar cells. (*g*) Transverse section of the circumoral nerve ring showing immunostained cells in the hyponeural and ectoneural regions. Immunostained fibres are present in the ectoneural neuropile (asterisk) and are continuous with immunostained fibres in the basi-epithelial plexus of the adjacent peristomial membrane. (*h*) Higher magnification image of the boxed ectoneural region of (*g*), showing immunostained bipolar shaped cells (arrowheads) in the subcuticular epithelium. (*i*) Higher magnification image of the boxed hyponeural region of (*g*), showing immunostained roundish monopolar cells (arrows) and immunostained fibres in the adjacent ectoneural neuropile (asterisk). CONR, circumoral nerve ring; Ec, ectoneural region; Hy, hyponeural region; MN, marginal nerve; PM, peristomial membrane; THS, transverse hemal strand; RHS, radial hemal strand; RNC: radial nerve cord; TF, tube foot. Scale bars: 4.8 µm in (*h*); 8 µm in (*b*,*e*,*f*,*i*); 32 µm in (*a*), (*a*) inset, (*d*,*g*); 36 µm in (*c*).

**Figure 9.**
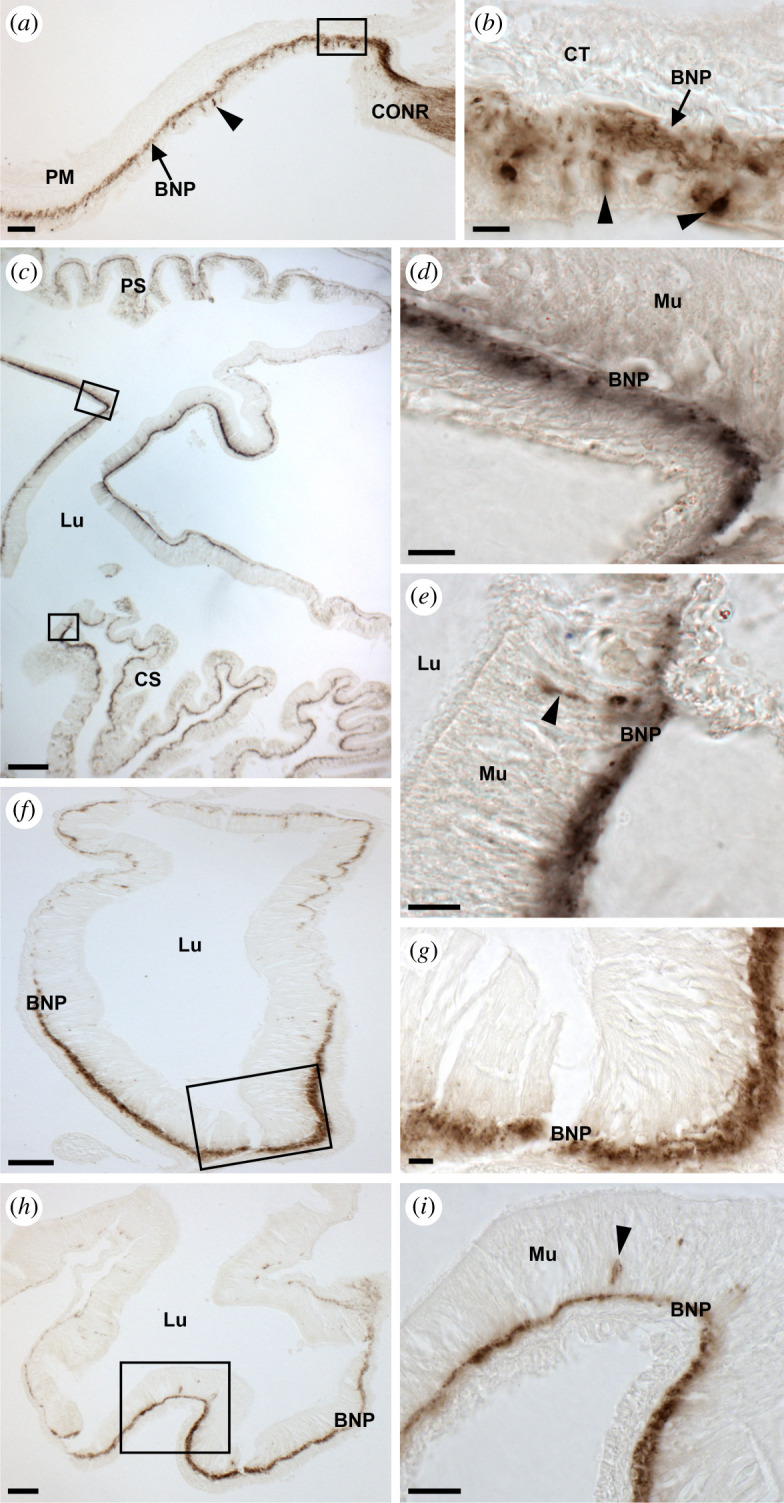
Localization of ArSS2 in the digestive system of *A. rubens* using immunohistochemistry. (*a*) Transverse section through the central disc showing ArSS2-immunoreactivity (immunostaining) in the peristomial membrane and the circumoral nerve ring. Immunostained cells are distributed sparsely in the external epithelial layer of the peristomial membrane and stained fibres are present in the underlying basi-epithelial nerve plexus and contiguous with the immunostained ectoneural region of the circumoral nerve ring. (*b*) Higher magnification image of the boxed region of peristomial membrane of (*a*), showing immunostained cells (arrowheads) in the external epithelial layer and immunostained fibres in the underlying basi-epithelial nerve plexus (arrow). (*c*) Transverse section through the central disc region showing immunostained fibres in the cardiac stomach and pyloric stomach regions. (*d*) Higher magnification image of the boxed region of the pyloric stomach in (*c*), showing immunostained fibres in the basi-epithelial nerve plexus. (*e*) Higher magnification image of the bottom boxed region of the cardiac stomach in (*c*), showing one bipolar shaped cell (arrowhead) in the mucosa layer and immunostained fibres in the basi-epithelial nerve plexus. (*f*) Transverse section of a pyloric duct showing immunostained fibres located on the aboral and oral sides, but with immunostaining more prominent on the oral side. (*g*) Higher magnification image of the boxed region of (*f*), showing immunostained fibres in the basi-epithelial nerve plexus on the oral side of the pyloric duct. (*h*) Transverse section of a starfish arm showing immunostaining present in a pyloric caecum, with region-specific variation in the intensity of immunostained fibres in the basi-epithelial nerve plexus. (i) Higher magnification image of the boxed region of the pyloric caecum in (*h*), showing immunostained cells (arrow) in the mucosal layer and immunostained fibres in the basi-epithelial nerve plexus. BNP, basi-epithelial nerve plexus; CONR, circumoral nerve ring; CS, cardiac stomach; CT, collagenous tissue; Lu, lumen; Mu, mucosa; PC, pyloric caecum; PD, pyloric duct; PM, peristomial membrane; PS, pyloric stomach. Scale bars: 12 µm in (*b*,*d*,*eg*); 24 µm in (*i*); 48 µm in (*a*), (*h*); 60 µm in (*f*); 120 µm in (*c*).

**Figure 10.**
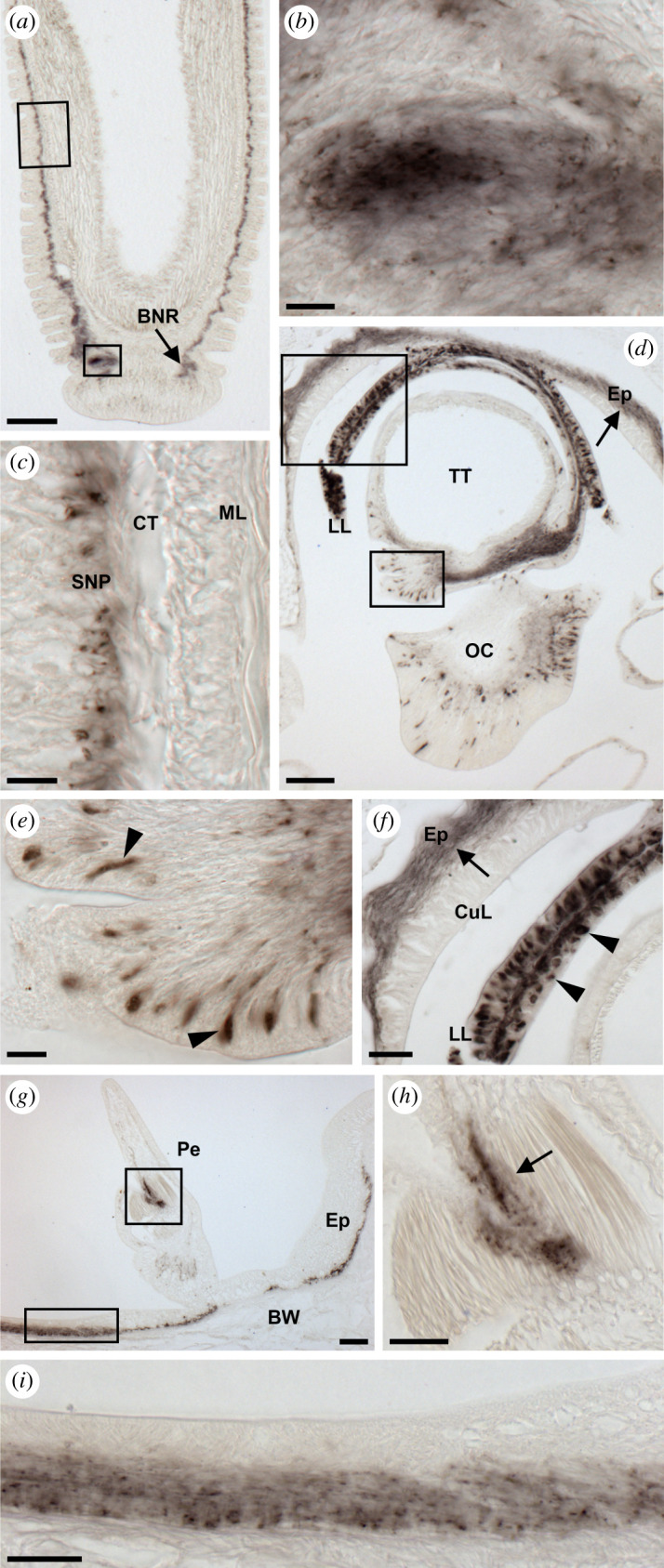
Localization of ArSS2 in the tube feet, body wall and terminal tentacle of *A. rubens* using immunohistochemistry. (*a*) Longitudinal section of a tube foot showing ArSS2-immunoreactivity (immunostaining) in the subepithelial nerve plexus along the length of the stem and extending into the basal nerve ring of the disc region. (*b*) Higher magnification image of the lower boxed region of (*a*), showing immunostaining in the basal nerve ring of the disc region. (*c*) Higher magnification image of the upper boxed region of (*a*), showing immunostaining in the subepithelial nerve plexus along the length of the stem. (*d*) Transverse section of an arm tip, showing immunostaining in the terminal tentacle, lateral lappet, optic cushion and the surrounding body wall epithelium (arrow). (*e*) High magnification image of the lower boxed region of (*d*), showing immunostained bipolar shaped cells in the folded external epithelial layer of the terminal tentacle (arrowheads). (*f*) High magnification image of the upper boxed region of (*d*), showing immunostained fibres in the basi-epithelial nerve plexus (arrow) of the body wall epithelium adjacent to the terminal tentacle and immunostained cells in a lateral lappet (arrowheads). (*g*) Transverse section of an arm showing immunostaining in a pedicellaria and the nerve plexus beneath the external epithelium of the body wall. (*h*) High magnification image of the upper boxed region of (*g*), showing immunostained fibres between muscles of a pedicellaria. (*i*) High magnification image of the lower boxed region of (*g*), showing immunostained fibres in the nerve plexus beneath the external epithelium of the body wall. BNR, basal nerve ring; BW, body wall; CT, collagenous tissue; CuL, cuticular layer; Ep, epithelium; LL, lateral lappet; ML, muscle layer; OC, optic cushion; Pe, pedicellaria; SNP, subepithelial nerve plexus; TT, terminal tentacle. Scale bars: 12 µm in (*b*,*c*,*e*); 24 µm in (*f*,*h*,*i*); 60 µm in (*d*,*g*); 120 µm in (*a*).

Informed by these results, the ArSS2 antiserum was used at a dilution of 1 : 5000 for immunohistochemical analysis of ArSS2 expression in *A. rubens*. ArSS2-immunoreactivity (ir) was revealed in the central nervous system (figure 8), digestive system (figure 9), tube feet and body wall (figure 10), with the distribution of immunostained cells consistent with distribution of cells expressing ArSSP2 transcripts (figures 5–7). No staining was observed in sections incubated with antiserum pre-absorbed with the ArSS2 peptide, confirming the specificity of immunostaining observed with the antiserum (figure 8*a* inset).

#### 3.7.1. Nervous system

ArSS2-ir was observed in the radial nerve cords (figure 8*a*,*b*,*d*–*f*), marginal nerves (figure 8*c*) and circumoral nerve ring (figure 8*g*–*i*) of *A. rubens*, with the location of stained cells consistent with the distribution of ArSSP2 transcripts revealed using mRNA *in situ* hybridization. Thus, in the radial nerve cords, bipolar shaped stained cells can be seen in the subcuticular epithelium of the ectoneural region (figure 8*a*,*b*,*d*,*e*) and roundish monopolar cells can be seen in the hyponeural region (figure 8*a*,*b*,*d*,*f*). In transverse sections, immunostained cells can be seen predominantly located in the lateral regions of the radial nerve cord and sparsely distributed in the apical region (figure 8*a*). Furthermore, immunostained fibres are present in the neuropile of the ectoneural region (figure 8*a*,*b*,*d*). However, the intensity of immunostaining in transverse sections of the ectoneural neuropile is not homogeneous, with some regions more densely stained than others (figure 8*a*). In longitudinal parasagittal sections of the radial nerve cord, it can be seen that stained cells in the ectoneural subcuticular epithelium are distributed along the length of the nerve cord without apparent clustering (figure 8*d*). Likewise, the intensity of immunostaining in the ectoneural neuropile appears to be quite homogeneous along the length of the nerve cord (figure 8*d*). This contrasts with the hyponeural region, where distinct clusters of stained cells can be seen in longitudinal sections, most notably in close proximity to the transverse hemal strand (figure 8*d*). The pattern of immunostaining observed in transverse sections of the circumoral nerve ring is consistent with that observed in transverse sections of the radial nerve cords, with stained cells in both the ectoneural and hyponeural regions and some variation in the density of immunostained fibres in the ectoneural neuropile (figure 8*g–i*). Immunostaining was also observed in the marginal nerves and internal to the marginal nerves immunostained fibres could be observed in the lateral motor nerves (figure 8*c*).

#### 3.7.2. Digestive system

Immunostaining was revealed in several regions of the digestive system in *A. rubens*, including the peristomial membrane (figure 9*a*,*b*), cardiac stomach (figure 9*c*,*e*), pyloric stomach (figure 9*c*,*d*), pyloric ducts (figure 9*f*,*g*) and pyloric caeca (figure 9*h*,*i*). The majority of this immunostaining is localized in the basi-epithelial nerve plexus of each region of the digestive system. In the peristomial membrane, the immunostained basi-epithelial nerve plexus is contiguous with the immunostained ectoneural region of the circumoral nerve ring (figure 9*a*,*b*). Immunostained cells are distributed sparsely in the external epithelial layer of the peristomial membrane (figure 9*a*,*b*). In the cardiac stomach, elongate shaped stained cells can be seen in the mucosal layer adjacent to the immunostained basi-epithelial nerve plexus (figure 9*e*). Transverse sections of the pyloric duct show that immunostaining is present throughout the basi-epithelial nerve plexus but the staining is most intense on the oral (lower) side of the pyloric duct (figure 9*f*,*g*). Likewise, regional variation in the intensity of immunostaining is observed in the basi-epithelial nerve plexus of the pyloric caeca (figure 9*h*,*i*).

#### 3.7.3. Tube feet, terminal tentacle and body wall

In longitudinal sections of tube feet, immunostaining can be seen in the subepithelial nerve plexus along the length of the podium and extending into the basal nerve ring of the disc region (figure 10*a–c*). Likewise, an extensive population of immunostained cells and an associated nerve plexus were revealed in the terminal tentacle and associated sensory organs, which include the optic cushion and lateral lappets (figure 10*d–f*). In the optic cushion, immunostained bipolar shaped cells were observed in the photoreceptor cell layer (figure 10*d*). Immunostaining was also observed in the nerve plexus beneath the external epithelium of the body wall and in nerve fibres innervating pedicellariae, which are pincer-like appendages that remove debris and encrusting material from the external body surface (figure 10*g–i*).

### 3.8. ArSS2 causes dose-dependent relaxation of tube foot and cardiac stomach preparations from *A. rubens*

**Figure 11.**
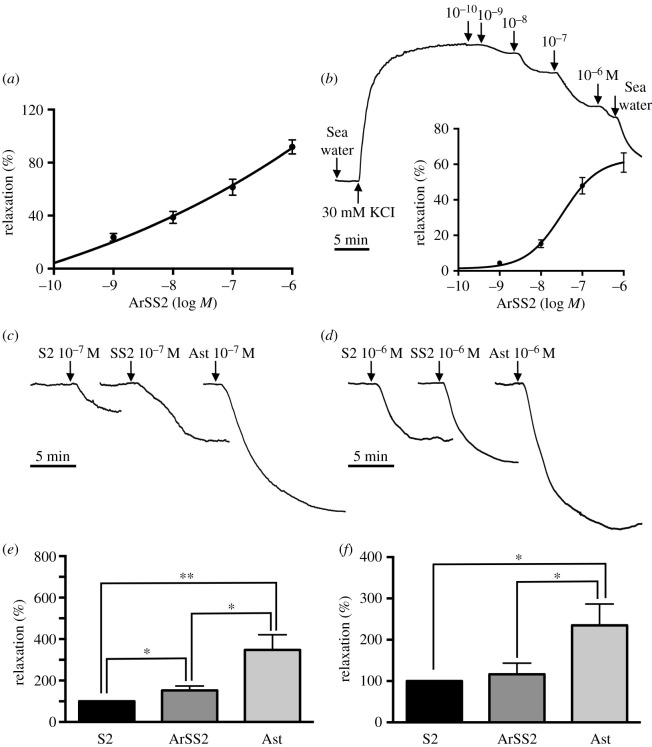
ArSS2 causes relaxation of *in vitro* preparations of tube feet and cardiac stomach from *A. rubens*. (*a*) Graphs showing the dose-dependent relaxing effect of ArSS2 on tube foot preparations at concentrations ranging from 10^−10^ to 10^−6^ M. The responses are expressed as the mean percentage reversal (±s.e.m.; *n* = 6) of the contraction induced by 10^−6^ M acetylcholine (ACh). (*b*) Upper: representative recording showing that ArSS2 causes dose-dependent relaxation of a cardiac stomach preparation that was pre-contracted with seawater containing 3 × 10^−2^ M added KCl. Lower: graph showing the dose-dependent relaxing effect of ArSS2 on cardiac stomach preparations at concentrations ranging from 10^−10^ to 10^−6^ M. The responses are expressed as the mean percentage reversal (±s.e.m.; *n* = 10) of the contraction induced by 3 × 10^−2^ M KCl. (c) Representative recording of a cardiac stomach preparation that compares the relaxing effects of ArSS2, the SALMFamide-type neuropeptide S2 and asterotocin (Ast) at a concentration of 10^−7^ M. As in (*b*), the cardiac stomach preparation was pre-contracted with KCl-supplemented seawater prior to application of the neuropeptides. The graph below compares the effects of S2, ArSS2 and Ast on cardiac stomach preparations at a concentration of 10^−7^ M, expressed as mean percentages (±s.e.m.; *n* = 6) with the relaxing effect of S2 defined as 100%. The relaxing effect of Ast is significantly larger than the effect of S2 (two-tailed Student's *t*-test; *p* = 0.0055; *n* = 6) and the effect of ArSS2 (two-tailed Student's *t*-test; *p* = 0.0250; *n* = 6). The relaxing effect of ArSS2 is significantly larger than the effect of S2 (two-tailed Student's *t*-test; *p* = 0.0303; *n* = 6). (*d*) Representative recording of a cardiac stomach preparation that compares the relaxing effects of S2, ArSS2 and Ast at a concentration of 10^−6^ M. As in (*c*), the cardiac stomach preparation was pre-contracted with KCl-supplemented seawater prior to application of the neuropeptides. The graph below compares the effects of S2, ArSS2 and Ast on cardiac stomach preparations at a concentration of 10^−6^ M, expressed as mean percentages (±s.e.m.; *n* = 6) with the relaxing effect of S2 defined as 100%. The relaxing effect of Ast is significantly larger than the effect S2 (two-tailed Student's *t*-test; *p* = 0.0255; *n* = 6) and the effect of ArSS2 (two-tailed Student's *t*-test; *p* = 0.0483; *n* = 6). There is not a significant difference in the magnitude of the relaxing effects of ArSS2 and S2.

*In vitro* pharmacological tests revealed that ArSS2 causes relaxation of tube foot and cardiac stomach preparations from *A. rubens*, but ArSS2 had no observable effects on the contractility of apical muscle preparations (data not shown). ArSS2 caused dose-dependent relaxation of tube foot preparations at concentrations between 10^−9^ and 10^−6^ M. At the highest concentration tested (10^−6^ M), ArSS2 caused 91% ± 13% (*n* = 6) reversal of ACh (10^−6^ M) induced tube foot contraction (figure 11*a*). Likewise, ArSS2 caused dose-dependent relaxation of cardiac stomach preparations at concentrations between 10^−9^ and 10^−6^ M. At the highest concentration tested (10^−6^ M), ArSS2 caused 60 ± 18% (*n* = 10) reversal of KCl (30 mM)-induced cardiac stomach contraction (figure 11*b*). Previous studies have revealed that both the SALMFamide neuropeptide S2 (SGPYSFNSGLTF-NH_2_) and the vasopressin/oxytocin-type neuropeptide asterotocin (CLVQDCPEG-NH_2_) act as cardiac stomach relaxants in *A. rubens* [64,67]. Therefore, to evaluate the efficacy of ArSS2 as a cardiac stomach relaxant, we compared its effect with the effects of S2 and asterotocin, testing the peptides at concentrations of 10^−7^ and 10^−6^ M. At 10^−7^ M, the mean magnitude of the relaxing action of ArSS2 was 1.5 times the effect of S2 but only 44% of the effect of asterotocin. The mean magnitude of the relaxing action of asterotocin was 3.5 times the effect of S2 (figure 11*c*), consistent with findings of a previous study [67]. At a concentration of 10^−6^ M, the mean magnitude of the relaxing action of ArSS2 was not significantly different to the effect S2 but was 50% of the effect of asterotocin (figure 11*d*). Thus, ArSS2 acts as a muscle relaxant in *A. rubens* but is less effective as a cardiac stomach relaxant than asterotocin.

### 3.9. ArSS2 triggers partial cardiac stomach eversion in *A. rubens*

**Figure 12.**
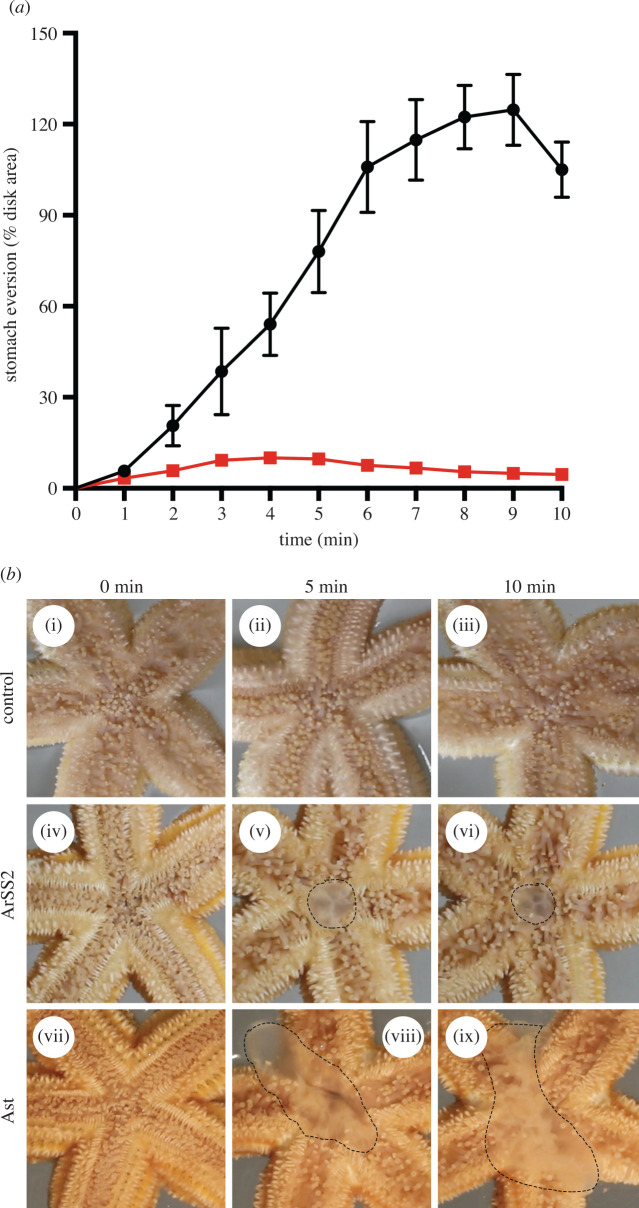
*In vivo* injection of ArSS2 triggers cardiac stomach eversion in *A. rubens*. (*a*) Temporal dynamics of ArSS2 (red; 10 µl of 10^−3^ M) and asterotocin (Ast) (black; 10 µl of 10^−3^ M) induced cardiac stomach eversion. The graph shows the mean area (±s.e.m.; *n* = 10) of the cardiac stomach everted expressed as a percentage of the area of the central disc region at 1 min intervals over a 10 min period following injection of ArSS2 or Ast. (*b*) Photographs from video recordings of the experiment in (*a*) showing representative control (water-injected) starfish (i–iii), ArSS2-injected starfish (iv–vi) and Ast-injected starfish (vii–ix) at 0, 5 and 10 min after injection. The cardiac stomach eversion area is labelled with a dashed line in (v,vi,viii,ix).

*Asterias rubens* feeds by everting its cardiac stomach through a narrow oral opening (mouth) and over the digestible parts of its prey, and for this to be accomplished, the cardiac stomach must be in a relaxed state [68,69]. Neuropeptides that cause cardiac stomach *in vitro* are therefore potential mediators of cardiac stomach eversion *in vivo*. Previous studies have revealed that injection of 100 µl 10^−3^ M asterotocin into the perivisceral coelom triggers complete eversion of the cardiac stomach in *A. rubens* [67]. Therefore, here we investigated if ArSS2 also triggers cardiac stomach eversion in *A. rubens*. Our *in vitro* experiments (figure 11) revealed that at a concentration 10^−6^ M, ArSS2 causes maximal relaxation of cardiac stomach preparations. The volume of the pervisceral coelomic fluid in *A. rubens* is approximately 10 ml in medium-sized animals, and therefore we injected 10 µl 10^−3^ M ArSS2 into the perivisceral coelom of *A. rubens* (*n* = 10) to achieve an estimated perivisceral concentration of 10^−6^ M *in vivo*. Starfish injected with 10 µl water were used as the control group (*n* = 5) and starfish injected with 10 µl 10^−3^ M asterotocin (*n* = 10) were used as a positive control group. Both ArSS2 and asterotocin triggered cardiac stomach eversion in all of the animals injected with these peptides, with eversion commencing at 1–2 min after injection (figure 12*a*). The mean maximal eversion occurred approximately 4 min after injection with ArSS2 and approximately 9 min after injection with asterotocin. However, the magnitude of cardiac stomach eversion induced by ArSS2 was much less than that induced by asterotocin, with the mean maximal eversion induced by ArSS2 and asterotocin corresponding to approximately 10% and 125% of the area of the central disc region, respectively. Representative images of starfish at 0, 5 and 10 min after injection with water, ArSS2 and asterotocin are shown in figure 12*b* (i–iii), (iv–vi) and (vii–ix), respectively.

## 4. Discussion

SS-type neuropeptides in vertebrates and allatostatin-C (ASTC)-type neuropeptides in protostome invertebrates (e.g. the arthropod *D. melanogaster*) are members of a family of structurally and evolutionarily related neuropeptides that originated in a common ancestor of the Bilateria [22,75]. Functional characterization of SS-type and ASTC-type neuropeptides has revealed that they typically act as inhibitory regulators of physiological processes in vertebrates and arthropods, respectively [7,76]. However, little is known about SS/ASTC-type neuropeptide signalling in other taxa. Of particular interest from an evolutionary perspective are deuterostome invertebrates, which occupy an ‘intermediate' phylogenetic position with respect to vertebrates and protostomes [45,47]. Accordingly, here we report the first molecular and functional characterization of SS/ASTC-type neuropeptide signalling in a deuterostome invertebrate—the starfish *A. rubens* (phylum Echinodermata).

### 4.1. Molecular characterization of SS/ASTC-type signalling in the starfish *A. rubens*

Analysis of *A. rubens* neural transcriptome sequence data enabled identification of two transcripts encoding SS/ASTC-type neuropeptide precursors (ArSSP1, ArSSP2), and the sequences of these precursors were confirmed by cDNA cloning and sequencing. Consistent with the characteristics of SS/ASTC-type precursors in other taxa [24], ArSSP1 and ArSSP2 each contain a single SS/ASTC-type neuropeptide located in the C-terminal region of the protein. Analysis of extracts of *A. rubens* radial nerve cords using mass spectrometry enabled investigation of the structure of the SS/ASTC-type neuropeptides derived from ArSSP1 and ArSSP2, which we refer to as ArSS1 and ArSS2, respectively. The structure of ArSS1 was determined as a 13-residue peptide (KCIGRFQPFSMPC) with a disulfide bridge between the two cysteine residues. Partial structural data were obtained for ArSS2 but a peptide with 4+ charged precursor mass 603.55 *m/z* and two cysteines modified by carbamidomethylation was detected (electronic supplementary material, figure S3b), consistent with the predicted structure of ArSS2 (RAKNARCMADFWKGRGLVDC, with a disulfide bridge between the two cysteine residues). Furthermore, synthesis and testing of this peptide revealed that it acts as a potent ligand for *A. rubens* SS/ASTC-type receptors, as discussed below.

Orthologues of both ArSSP1 and ArSSP2 are present in other echinoderms, including ophiuroids (brittle stars), echinoids (e.g. sea urchins) and holothurians (sea cucumbers) [48,49,77]. Furthermore, the predicted neuropeptides derived from these precursor proteins share sequence similarity with ArSS1 and ArSS2. A characteristic of the echinoderm SS-like peptides and SS/ASTC-type neuropeptides in other taxa is a pair of cysteine residues, which form a disulfide bridge in the mature peptides. However, there is variability in the number of residues between the cysteine residues (figure 1), with echinoderm SS1-type peptides having 10 residues, echinoderm SS2-type peptides having 12–15 residues, human SS/CST-type peptides having 10 residues, an SS-type peptide in the invertebrate chordate *Branchiostoma floridae* having 13 residues and protostome ASTC-type peptides having 6 residues. Thus, in this respect, the echinoderm SS-like peptides share more similarity with chordate SS/CST-type peptides than with protostome ASTC-type peptides. However, more specific sequence comparisons revealed that echinoderm SS1-type peptides have a core FXP motif (where X is variable) in common with ASTC-type neuropeptides from *D. melanogaster* and *C. elegans*, whereas the echinoderm SS2-type peptides have a core FWK/IWK motif in common with chordate SS-type neuropeptides. This suggests that echinoderm SS1-type peptides may be orthologues of protostome ASTC-type neuropeptides and echinoderm SS2-type peptides may be orthologues of chordate SS-type neuropeptides.

Previous studies have revealed that the presence, position and phase of introns are evolutionarily conserved characteristics of neuropeptide precursor genes that can provide evidence of orthology [78]. Here, we found that, in common with SS/ASTC-type genes in other taxa, echinoderm SS1-type and SS2-type precursor genes have a phase 0 intron at a position that interrupts the coding sequence for the N-terminal part of the precursor proteins. Furthermore, echinoderm SS1-type precursor genes have a second intron that interrupts the coding sequence for the C-terminal half of the precursor proteins, which is also a feature of the *C. elegans* and *D. melanogaster* ASTC genes (figure 2). However, this could be a convergent characteristic due to differences in the phase of the second intron. Thus, analysis of gene structure provided additional evidence that echinoderm SS1-type and SS2-type neuropeptides share a common evolutionary origin with SS/ASTC-type neuropeptides in other taxa, but without providing definitive evidence of a more specific orthology relationship with protostome ASTC-type and chordate SS-type neuropeptides, respectively.

To further investigate relationships of echinoderm SS/ASTC-type neuropeptides with SS/ASTC-type neuropeptides in other taxa, we sought to identify receptors for the *A. rubens* neuropeptides ArSS1 and ArSS2. Three *A. rubens* G-protein-coupled receptors (ArSSR1, ArSSR2 and ArSSR3) that are homologues of SS/ASTC-type receptors in other taxa were identified. Furthermore, homologues of these receptors were also identified in other echinoderms and phylogenetic analysis revealed that the *A. rubens* and other echinoderm receptors are positioned in a clade comprising chordate SS-type receptors, chordate opioid receptors and protostome ASTC-type receptors. Importantly, this clade is distinct from clades comprising MCH-type receptors and UII-type receptors. Therefore, ArSSR1–3 and related receptors in other echinoderms can be classified as SS/ASTC-type receptors.

Pharmacological characterization of ArSSR1–3 revealed that ArSS2 acts as a ligand for all three receptors, but the efficacy and potency of ArSS2 as a ligand was higher for ArSSR1 and ArSSR2 than for ArSSR3. Importantly, ArSS1 did not act as a ligand for any of the receptors. Thus, ArSS2 and ArSSR1–3 in *A. rubens* were identified here as molecular components of the first SS/ASTC-type neuropeptide signalling system to be characterized pharmacologically in an echinoderm. The identity of the receptor(s) for ArSS1 and SS1-type neuropeptides in other echinoderms remains to be determined. It is noteworthy, however, that our analysis of echinoderm genome sequence data has not identified any additional candidate SS/ASTC-type receptors; for example, BLAST analysis of the genome sequence of the starfish *A. planci* only identified genes encoding orthologues of the three *A. rubens* receptors (ArSSR1–3) that we have characterized here as receptors for ArSS2. We speculate, therefore, that ArSS1 and orthologues of ArSS1 in other echinoderms may act via receptors that are distinct from the family of SS/ASTC-type GPCRs (including ArSSR1–3) that have been characterized in bilaterians. Further studies will be needed to address this issue.

### 4.2. Functional characterization of ArSS2 in the starfish *A. rubens*

Having identified ArSS2 as the ligand for three SS/ASTC-type receptors in *A. rubens*, we then investigated the expression and pharmacological actions of ArSS2 to obtain the first insights into the physiological roles of a SS/ASTC-type neuropeptide in a deuterostome invertebrate. The use of mRNA *in situ* hybridization and immunohistochemistry revealed that cells/fibres expressing ArSSP2 transcripts and ArSS2, respectively, are present in the central nervous system, digestive system and the body wall and its associated appendages (e.g. tube feet) of *A. rubens*. This pattern of expression can be interpreted with reference to knowledge of the anatomy of the starfish nervous system [71,79] and in comparison with the distribution of other neuropeptides in *A. rubens* [63,67,72,80–84]. Thus, the presence of ArSS2 in the various organ systems of *A. rubens* is broadly similar to other neuropeptides, but there are specific local differences in patterns of neuropeptide expression.

In common with other neuropeptides (e.g. the calcitonin-type neuropeptide ArCT [83]), ArSS2 is expressed by neurons in both the ectoneural region (containing sensory and interneurons) and the hyponeural region (containing motoneurons) of the radial nerve cords and circumoral nerve ring, which contrasts with neuropeptides that are only expressed in the ectoneural region (e.g. the vasopressin/oxytocin-type neuropeptide asterotocin) [67]. Based on this pattern of expression, we can infer that ArSS2 acts as a signalling molecule in the ectoneural region of the central nervous system in *A. rubens,* but further studies will be required to determine more specifically its roles. Furthermore, the expression of ArSS2 in hyponeural motoneurons is indicative of roles in neuromuscular signalling.

ArSS2 was detected in cells and processes in body wall-associated muscular organs that enable locomotion in starfish (tube feet) and in muscle-containing regions of the digestive system (cardiac stomach). These findings provided a basis for testing ArSS2 as a potential myoactive neuropeptide. ArSSP2 transcripts are detected in cells located along the length of tube foot podia and ArSS2-immunoreactivity is present in the subepithelial nerve plexus of tube foot podia. Consistent with this expression of ArSS2 in tube feet, *in vitro* pharmacological tests revealed that ArSS2 causes dose-dependent relaxation of tube foot preparations. Furthermore, expression of ArSS2 in the disc region of tube feet is of interest because of its role in secreting proteins that enable attachment to and detachment from the substratum [85]. Thus, ArSS2 and other neuropeptides expressed in the disc region [67,80–83] may be involved in neural control of the secretion of these proteins. Consistent with the expression of ArSS2 in the tube feet, an extensive population of immunostained cells and fibres were revealed in the tube foot-like sensory terminal tentacle located at the tip of each in arm and in its associated sensory organs, which include lateral lappets (presumptive olfactory organs) and the optic cushion (simple eye) [86,87]. Interestingly, there is evidence that SS/ASTC-type neuropeptides are evolutionarily ancient regulators of circadian activity [32,88,89] and therefore the expression of ArSS2-expressing cells in the photoreceptor cell layer of the optic cushion in *A. rubens* may be evidence of this role in starfish.

ArSS2 is widely expressed in the digestive system of *A. rubens*, including the peristomial membrane, cardiac stomach, pyloric stomach, pyloric duct, and pyloric caeca. In the cardiac stomach, ArSSP2 transcripts are detected in cells located in the mucosa layer and ArSS2-immunoreactivity is present in the adjacent basi-epithelial nerve plexus. Consistent with this expression of ArSS2 in the cardiac stomach, ArSS2 causes dose-dependent relaxation of cardiac stomach preparations. Other neuropeptides that cause relaxation of tube foot and/or cardiac stomach preparations have been identified in *A. rubens*, which include the SALMFamide neuropeptides S1 and S2 [64,66], pedal peptide/orcokinin-type neuropeptides [81,82], the calcitonin-type neuropeptide ArCT [83] and the vasopressin/oxytocin-type neuropeptide asterotocin [67]. Therefore, using the cardiac stomach as a test preparation, we compared the efficacy of ArSS2 as a cardiac stomach relaxant with two of these neuropeptides—S2 and asterotocin. This revealed that at a concentration of 10^−7^ M, the efficacy of ArSS2 was 1.5 times greater than that of S2, but only 44% of the effect of asterotocin. At 10^−6^ M, there was no significant difference in the efficacy of ArSS2 and S2, but the efficacy of these peptides was approximately 50% of that of asterotocin (figure 11). A previous study revealed that asterotocin is much more effective as a cardiac stomach relaxant than S2 [67] and here, we show that asterotocin is more effective than both S2 and ArSS2 as a cardiac stomach relaxant when tested at 10^−7^ or 10^−6^ M.

The *in vitro* relaxing effect of ArSS2 on the cardiac stomach of *A. rubens* is of interest from a physiological/behavioural perspective because relaxation of the cardiac stomach is required for the extraoral feeding behaviour of starfish where it is everted out of the mouth and over the digestible soft tissue of prey such as mussels [68,69]. Therefore, here, we investigated the effect of *in vivo* injection of ArSS2 in *A. rubens* and found that it causes partial eversion of the cardiac stomach. Our previous studies have revealed that both S2 and asterotocin also cause cardiac stomach eversion in *A. rubens* but, consistent with their relative efficacy as cardiac stomach relaxants *in vitro*, asterotocin is much more effective and potent than S2 in triggering cardiac stomach eversion [67]. Accordingly, here, we found that asterotocin is much more effective than ArSS2 in triggering cardiac stomach eversion, consistent with their relative effectiveness as cardiac stomach relaxants *in vitro*. Nevertheless, we conclude that ArSS2 is one of several neuropeptide types that may participate in physiological mechanisms associated with control of cardiac stomach eversion during feeding in *A. rubens*.

Finally, it should be noted that ArSS2 did not cause relaxation when tested on preparations of the apical muscle, which is located along the aboral coelomic lining of starfish arms. Accordingly, we did not detect expression of ArSS2 in the apical muscle. These findings are consistent with our results from testing other neuropeptides in *A. rubens*, which have revealed that while some neuropeptides cause relaxation of cardiac stomach, tube foot and apical muscle preparations (e.g. the pedal peptide-type neuropeptide ArPPLN1b [81]), other neuropeptides cause relaxation of only one or two of these preparations. For example: (i) the vasopressin-type neuropeptide asterotocin caused relaxation of cardiac stomach and apical muscle preparations but not tube feet [67]; (ii) the calcitonin-type neuropeptide ArCT caused relaxation of apical muscle and tube foot preparations but not cardiac stomach [83]; (iii) the pedal peptide-type neuropeptide ArPPLN2 h caused relaxation of cardiac stomach preparations but not tube feet or apical muscle [82]; and (iv) the luqin-type neuropeptide ArLQ caused relaxation of tube foot preparations but not cardiac stomach or apical muscle [63]. Further insights into the physiological significance of these differences in the actions of neuropeptides that act as muscle relaxants in starfish may be obtained by detailed comparative analysis of the expression of the receptors that mediate their effects.

### 4.3. Evolutionary and comparative physiology of SS/ASTC-type neuropeptide signalling in the Bilateria

Our discovery that ArSS2 acts as a muscle relaxant in *A. rubens* is noteworthy because it provides the first insight into the physiological roles of SS-type neuropeptides in a deuterostome invertebrate. A common theme emerging from investigations of the physiological roles of SS-type neuropeptides in vertebrates and ASTC-type neuropeptides in protostome invertebrates is that they typically act as inhibitory regulators of physiological processes at the level of cells, tissues or organs. For example, SS inhibits gut motility [8,9] and hormone secretion [1] in mammals and ASTC inhibits cardiac and gut activity [30,33] and hormone synthesis [23] in insects. Therefore, our discovery that an SS-type neuropeptide (ArSS2) acts as a myoinhibitory agent (muscle relaxant) in an echinoderm (*A. rubens*) is consistent with the inhibitory actions of SS/ASTC-type neuropeptides in other taxa. Accordingly, our findings combined with previous findings from vertebrates and arthropods indicate that the role of SS/ASTC-type neuropeptides as inhibitory regulators of physiological processes can be traced back to the urbilaterian common ancestor deuterostomes and protostomes. However, while the actions of SS/ASTC-type neuropeptides at the cell/tissue/organ level are typically inhibitory, at a systems/behavioural level, this may manifest as an inhibitory or a stimulatory action. For example, injection of an ASTC-type peptide inhibits foregut motility, food intake and growth in larval tomato moth *L. oleracea* [33]. By contrast, SS stimulates food intake and drinking in mammals, which involves complex interactions with other neuronal signalling systems in the brain that regulate feeding/drinking [90]. Accordingly, the inhibitory action of ArSS2 in causing cardiac stomach relaxation may manifest at a behavioural level in triggering the dynamic process of cardiac stomach eversion when starfish feed.

## 5. Conclusion

We have obtained the first insights into the molecular properties and physiological roles of SS-type neuropeptide signalling in a deuterostome invertebrate—the starfish *A. rubens* (phylum Echinodermata). Discovery of an SS-type signalling system in *A. rubens* comprising ArSS2 and its cognate receptors ArSSR1–3 provides a missing link in our knowledge of the molecular evolution of SS/ASTC-type signalling in the Bilateria. Our finding that ArSS2 acts as a muscle relaxant in *A. rubens* provides new evidence that SS/ASTC-type neuropeptides have an evolutionarily ancient and conserved role as inhibitory regulators of physiological processes in the Bilateria. Furthermore, the *in vivo* effect of ArSS2 in triggering stomach eversion in *A. rubens* is indicative of an evolutionarily ancient and conserved role of SS/ASTC-type neuropeptides as regulators of feeding behaviour. However, given the widespread expression of ArSS2 in *A. rubens* in the central nervous system and peripheral organ systems, it is likely that ArSS2 is a pleiotropic peptide and also has other roles. For example, the expression of ArSS2 by neurons in the ectoneural region of the radial nerve cords and circumoral nerve ring is indicative of roles as a neuromodulator and here parallels can be drawn with the expression of SS in subpopulations of inhibitory GABAergic neurons in cortical regions of the mammalian brain [10,11]. Furthermore, as highlighted above, the expression of ArSS2 by cells in the photoreceptor cell layer of the optic cushion in *A. rubens* may be indicative of roles associated with regulation of circadian activity. Clearly, further investigation of the actions of ArSS2 in *A. rubens* will be required to obtain broader insights into its physiological roles. Furthermore, although the receptor(s) that mediates the effects of ArSS1 remains to be identified, it will also be of interest to compare the expression and actions of ArSS1 and ArSS2 in *A. rubens*. Likewise, investigation of the actions of both SS1-type and SS2-type neuropeptides in sea cucumbers, brittle stars and sea urchins would provide a comparative perspective on the physiological roles of SS/ASTC-type neuropeptides in other echinoderms.

## Supplementary Material

Electronic Supplementary Material

## Data accessibility

*Asterias rubens* radial nerve cord transcriptome sequence data generated using Illumina HiSeq has been deposited in the NCBI Sequence Read Archive (SRA) (SRP068147; http://www.ncbi.nlm.nih.gov/sra/SRP068147). Novel nucleotide sequences reported in this paper have been deposited in the GenBank database under accession numbers MN257487 (ArSSP2), MN251831 (ArSSR1), MN251832 (ArSSR2) and MN251833 (ArSSR3). The novel rabbit antiserum to ArSS2 has been assigned the RRID: AB_2847910. The datasets supporting this article have been uploaded as part of the electronic supplementary material.
