# Supplementary material for: Molecular and functional characterization of somatostatin-type signalling in a deuterostome invertebrate
Source: Open Biol. 2020 Sep 9;10(9):200172. doi: 10.1098/rsob.200172 (PMC7536072; doi:10.1098/rsob.200172)
Supplement: Electronic Supplementary Material [file rsob200172supp1.pdf]

ELECTRONIC SUPPLEMENTARY MATERIAL

Molecular and functional characterisation of somatostatin-type signalling in a deuterostome invertebrate

Ya Zhang[1], Luis Alfonso Yañez Guerra[1], Michaela Egertová[1],

Cleidiane G. Zampronio[2], Alexandra M. Jones[2], Maurice R. Elphick[1*]

[1]School of Biological and Chemical Sciences, Queen Mary University of London, E1 4NS

[2]School of Life Sciences and Proteomics Research Technology Platform, University of Warwick, Coventry, CV4 7AL, UK

Open Biology, https://royalsocietypublishing.org/doi/10.1098/rsob.200172

```
1    cttaacatcagcttcatggaattgggctctagtgatcgtttgatatgtttaccctaagtg
61   aatagcctattaatatcgttttcttcttttaaatgttgtttgaagatttaaccgtgacaa
121  attgatatgcaattgaaaaaatcatcacccaatctcttggtctgtggctctctattttga
181  gatgcaagtgagcgtgcaattgtctattgatgaggagcagagtttcgttccgctccctga
241  tacagaccactgtagcaagccaacccccagctctcatcactgaaaccaaaccactgagat
301  cacccaatccattttaaaacgtgcacttcgtgccagcgtggtatttaaaggttcactcaa
361  cagttggtgccatttccgaacccagccacaggcctttgagaagagaagaggcggtcttaa
421  ctgcgcgcccatgcccacaatccagtgatcacatcttgtcaccctcttacatcaactact
481  cgatacgcgttataggaaacatctcgcgatcgctcatgtctctctccctcccccatacgg
541  cactcactcaaccacgccgtttgaccattgaaatgacgcactgacgacatcgtgggctac
601  aacagtttcttaagcagcgcgctccggaggttcacacatgggcggttacttcttctccaa
661  gcaggtccactgtacatcgactcgactggcgcggcgctaaaccatagactccattgctag
721  gaagaaacccgtggatgcagagttatttgtgagttatacttgcgctccaactcaaaaact
781  cgacaaaataataagtgaagacaagaaaaaagggggttacgtggagcgcccattcctcc
841  tcctggagtcttcttaaaggggccttggtttttagtttcgtgaaactctacttcttta
901  aacaccatgaggttctgttgtgttgtagtggttctaccgttgatctgtgtgctagctggg
               M  R  F  C  C  V  V  V  V  L  P  L  I  C  V  L  A  G   18
961  tgcctagtggcccatgcagcaccgaggaggggcggaggaggcaacagcgacccgcggtgg
      C  L  V  A  H  A  A  P  R  R  G  G  G  G  N  S  D  P  R  W   38
1021 aaaaggaatttctcgccaccgggaatgcagagctcgggcggcagctacaataaaggagat
      K  R  N  F  S  P  P  G  M  Q  S  S  G  G  S  Y  N  K  G  D   58
1081 cttgttgaaaggatattgaacagattacaagaacgtctcctcgggaaagttgacctctct
      L  V  E  R  I  L  N  R  L  Q  E  R  L  L  G  K  V  D  L  S   78
1141 caaaccaacacatggcatggaaatcagagtccaaaagaattagaccttcaacgatatagt
      Q  T  N  T  W  H  G  N  Q  S  P  K  E  L  D  L  Q  R  Y  S   98
1201 gatcaagaggatgagtttatagacgatgatgatgaagtgcctaaccgcccggctattaag
      D  Q  E  D  E  F  I  D  D  D  D  E  V  P  N  R  P  A  I  K  118
1261 aggaaatgtatcggtcggtttcaacctttctctatgccctgttagagttcacccagccta
      R  K  C  I  G  R  F  Q  P  F  S  M  P  C  *                 132
1321 tcagggctgtttggtctcataagaacctcccgtacaagattattttaagggctgtgttt
1381 tgaaattgagcgtctttggctatggctgatgacgtc
```

Supplementary Figure 1. *Asterias rubens* somatostatin-type precursor 1 (ArSSP1). The nucleotide sequence (lowercase, numbered on the left) encoding ArSSP1 (uppercase, numbered on the right) is shown. The predicted signal peptide is highlighted in blue, a putative dibasic cleavage site is highlighted in green and the ArSS1 neuropeptide sequence is highlighted in red. The asterisk shows the position of the stop codon. This sequence was determined from contig 1107850 of an *A. rubens* radial nerve cord transcriptome and has been deposited in GenBank under accession number KT601708. A cDNA containing the coding region was cloned by PCR using specific primers (underlined) and sequenced.

```
1    ccccgttccggagcaagttaaaaagccacgaccaagcgggagatgcttcattgataacgc
61   aatcacaggcagaacacgacaattcgttaagtgacaacgatcgctcgcatcagagacaca
121  cacacggagaaaagcagacgacattttaaccgagaaggccaacgcaaaagcatagacgt
181  cgttcccgcttctttttatacacaaacattccttttctctttgcaaagatgaatgcctcg
                                                     M  N  A  S     4
241  cctatgatgagccttgttgtggtcgcactctgcgccctactggtcgtctgtcaagctgac
      P  M  M  S  L  V  V  V  A  L  C  A  L  L  V  V  C  Q  A  D   24
301  ctcacagatataggaaacagcgacataatgaatgaacctggatcggaaaacggagggttc
      L  T  D  I  G  N  S  D  I  M  N  E  P  G  S  E  N  G  G  F   44
361  cttcatttcttgagagaggacctggtcccaagatacctcctcagacaggtggcatgaaa
      L  H  F  L  R  E  D  L  V  P  R  Y  L  L  R  Q  G  G  M  K   64
421  aagcgaccattcagcaacagcaatgacatggaccccatctcagaagtcaagaggagagcg
      K  R  P  F  S  N  S  N  D  M  D  P  I  S  E  V  K  R  R  A   84
481  aagaatgcaaggtgtatggctgacttctggaagggcagaggactggttgactgctagatg
      K  N  A  R  C  M  A  D  F  W  K  G  R  G  L  V  D  C  *     104
541  atcatgtctcatatcggccatcgtacataactggatccagttcaaaccagatcggagcgg
601  atcaaatcagatggcaccggactttttgacaacagtggactttttttgacgccgatttttg
661  tttgtaaagttgctgtatatatttagatcggaaaccccgttttaaaggaaggaaatgtga
721  ttatttagtcgggtctgattccatagtgaaaacacaatggtacaatgaccccgggtaaat
781  cttaagtctgaacaaaaatagccggggtcctttgagattcccctaaatggacggacatca
841  gcgagtttctttaaagtttgaatgccgtcatctcactcttcatttccccttaaatggaac
901  caattttgactcttctacatccatcggatattgcacataataaagcgatattccacccat
961  cactttcagcaaagcatttctgacttctgatcacacgtcatcggtgacgtcatcggttaa
1021 tttatgttggtttactgattctgcacatttattttaaggggtcgtagagtcatccacacc
1081 cggagtcttgagcaccgttgtctgtttgacaaatgacccttcaaatgcaccgtaggctta
1141 gaagtaacaattttttagaataagctgcatgaaattcaattgacattactagattgtgaag
1201 acgtttattgaggattctcaccaatcaaaccccaagccaagaaataattgtacaaacatc
1261 tcgttacaggttctagtgtatagagtctgtgatcttaaaggagtctagcgtgtgtgtatg
1321 gtcgtagaaggattaccacaaattacttgaccgactcacattgaccaatgtaaagtgttg
1381 ggcaggaatcgtgaagaaaaggggcatgctgattacaaacgattgacacatatgtttgg
1441 tgattaacggaagtttcctcaaaaacacttgcaaagtttgtacaataatataaagcaccg
1501 aagtgcttacaatgggaaaacacatgtatacaaaacaatgagacgccaagtccgtgcgtc
1561 agtttttttttt
```

Supplementary Figure 2. *Asterias rubens* somatostatin-type precursor 2 (ArSSP2). The nucleotide sequence (lowercase, numbered on the left) encoding ArSSP2 (uppercase, numbered on the right) is shown. The predicted signal peptide is highlighted in blue, a putative dibasic cleavage site is highlighted in green and the ArSS2 neuropeptide sequence is highlighted in red. The asterisk shows the position of the stop codon. This sequence was determined from contig 1111056 of an *A. rubens* radial nerve cord transcriptome and has been deposited in GenBank under accession number MN257487. A cDNA containing the coding region was cloned by PCR using specific primers (underlined), sequenced and then used as template for generating probes for localisation ArSSP1 expression in *A. rubens* using mRNA *in situ* hybridisation.

(a)

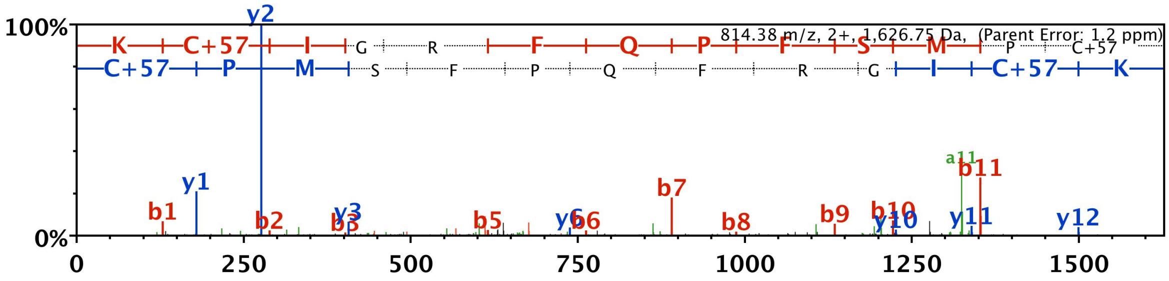

(b)

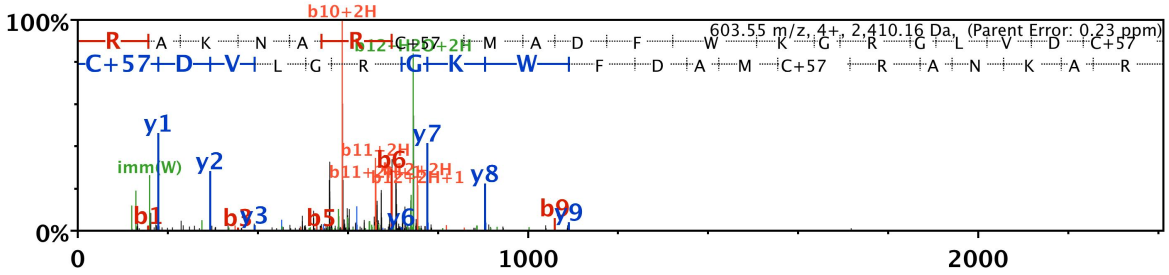

Supplementary Figure 3. Mass spectrometric characterisation of neuropeptides (ArSS1 and ArSS2) derived from ArSSP1 and ASSP2 in extracts of *A. rubens* radial nerve cords. **(a)** MS/MS HCD data for a 2+ charged precursor ion 814.38 m/z for the peptide ArSS1 derived from reduced and alkylated samples of radial nerve cord extract. **(b)** MS/MS HCD data for a 4+ charged precursor ion 603.55 m/z for the peptide ArSS2 derived from reduced and alkylated samples of radial nerve cord extract. The b series of peptide fragment ions are shown in red, the y series in blue and additional identified peptide fragment ions in green. The amino acid sequence identified in the mass spectrum is highlighted above the figures. C+57 represents cysteine modified by carbamidomethylation.

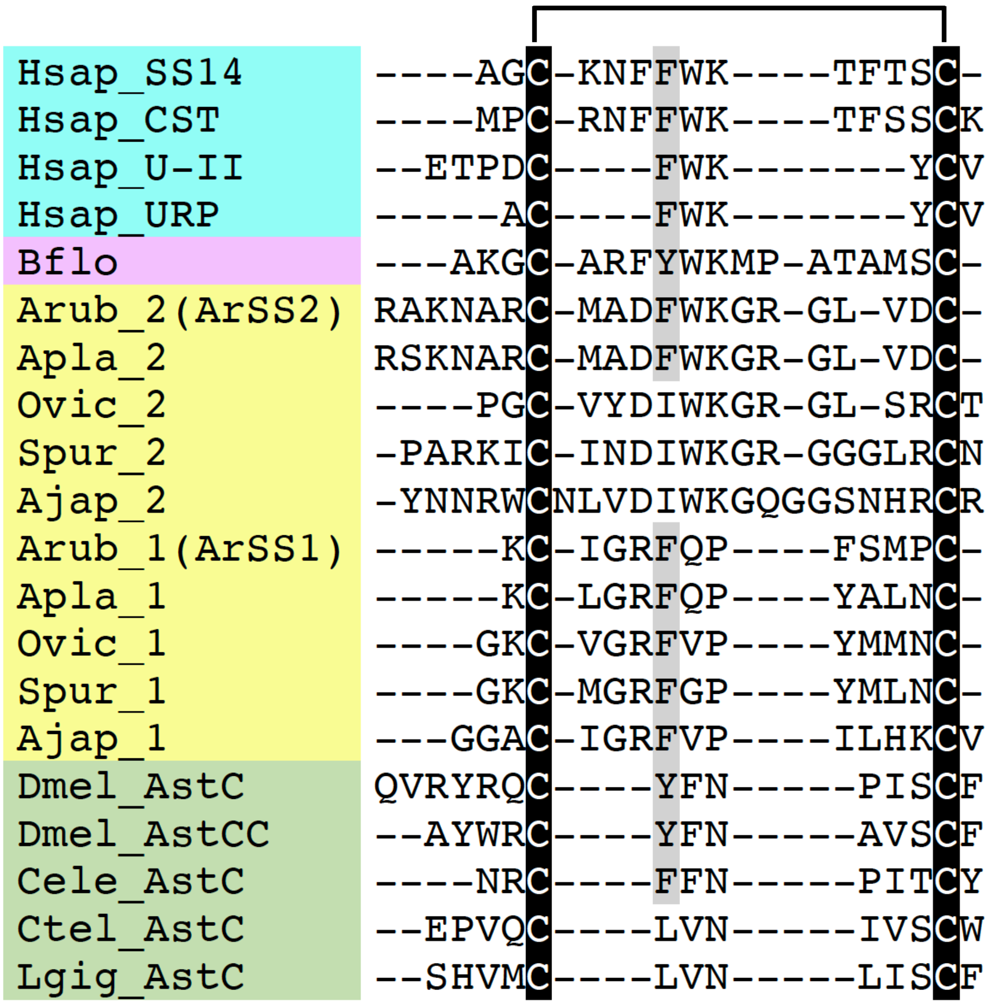

Supplementary Figure 4. Alignment of echinoderm somatostatin(SS)/allatostatin-C(ASTC)-like neuropeptides with chordate SS-type neuropeptides and protostome ASTC-type neuropeptides. The aligned amino acids are highlighted in black if the residue is present in at least 70% of the sequences or highlighted in grey if conservative amino acid substitutions are present in at least 70% of the sequences. The position of a disulphide bridge between the two conserved cysteine residues is shown above the alignments. Species and peptide names are highlighted in taxon-specific colours: blue (Chordata), pink (Hemichordata), yellow (Echinodermata), green (Protostomes). Species name abbreviations are as follows: Ajap (*Apostichopus japonicus*), Apla (*Acanthaster planci*), Arub (*Asterias rubens*), Bflo (*Branchiostoma floridae*), Cele (*Caenorhabditis elegans*), Ctel (*Capitella teleta*), Dmel (*Drosophila melanogaster*), Hsap (*Homo sapiens*), Lgig (*Lottia gigantea*), Ovic (*Ophionotus victoriae*), Spur (*Strongylocentrotus purpuratus*).

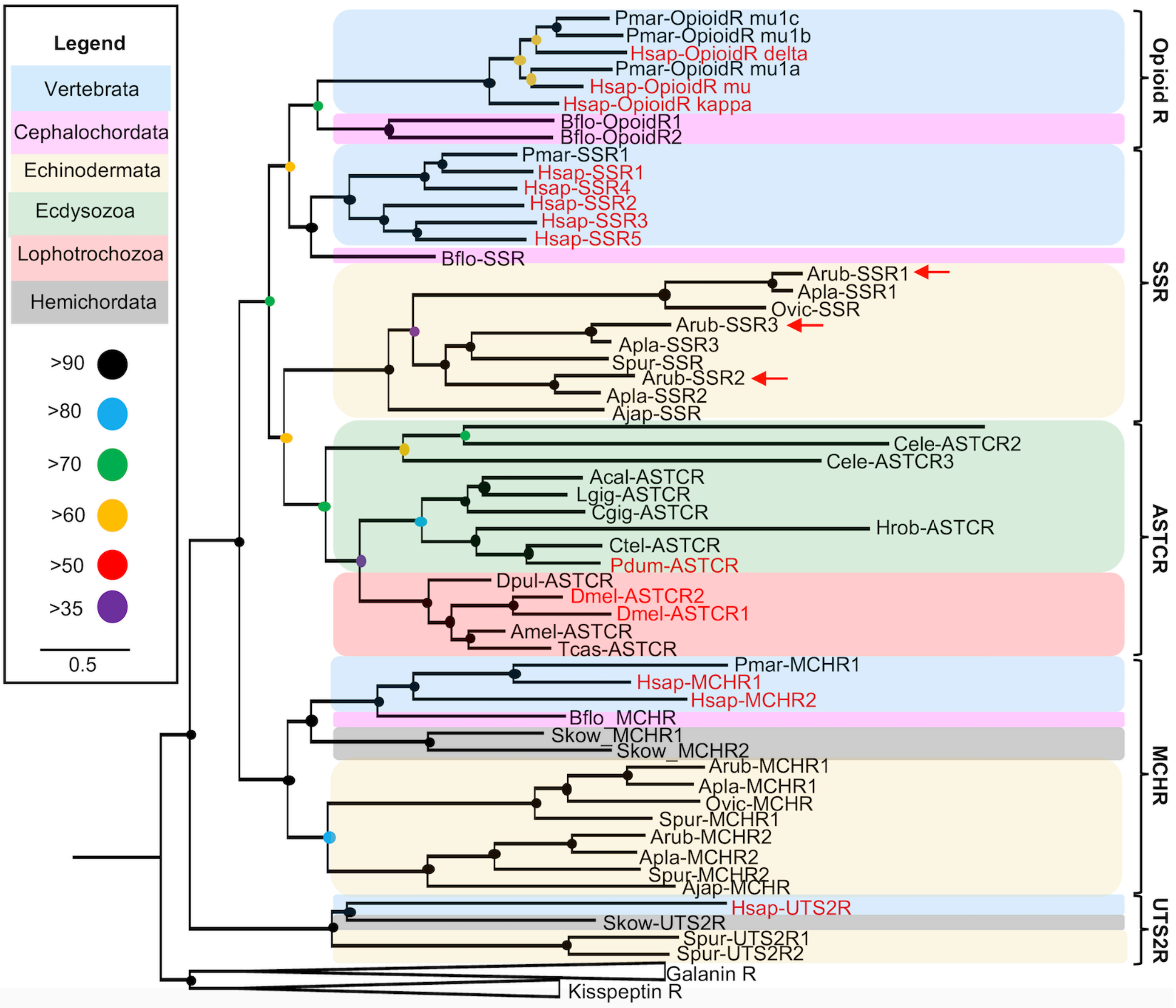

Supplementary Figure 5. Phylogenetic analysis identifies three *A. rubens* receptor proteins as somatostatin/allatostatin-C-type receptors. The tree generated using the maximum-likelihood method (W-IQ-tree online version 1.0) comprises three distinct receptor clades - SS/opioid/ASTC -type receptors, MCH-type receptors and urotensin II-type receptors. Galanin receptors and kisspeptin receptors were included here as outgroups to root the tree. The three *A. rubens* receptor proteins characterised in this study (labelled with red arrows) are positioned in the clade containing SS/opioid/ASTC receptors, demonstrating that they can be classified as SS/ASTC-type receptors. The round dots represent bootstrap support (1000 replicates) and the different coloured backgrounds represent different taxonomic groups (see legend). The scale bar represents the average residue substitution per site. Receptor names shown in red indicate that cognate ligands for these receptors have been identified experimentally. Species names are as follows: Acal (*Aplysia californica*), Ajap (*Apostichopus japonicus*), Amel (*Apis mellifera*), Apla (*Acanthaster planci*), Arub (*Asterias rubens*), Bflo (*Branchiostoma floridae*), Cele (*Caenorhabditis elegans*), Cgig (*Crassostrea gigas*), Ctel (*Capitella teleta*), Dmel (*Drosophila melanogaster*), Dpul (*Daphnia pulex*), Hrob (*Helobdella robusta*), Hsap (*Homo sapiens*), Lgig (*Lottia gigantea*), Ovic (*Ophionotus victoriae*), Pdum (*Platynereis dumerilii*), Pmar (*Petromyzon marinus*), Skow (*Saccoglossus kowalevskii*), Spur (*Strongylocentrotus purpuratus*), Tcas (*Tribolium castaneum*). Accession numbers of receptor sequences and associated references used to generate this figure are listed in Supplementary Table 4.

```
1                                                                  gt
3    ccgctgacaggaactacaattctatatctcgcaacactgtgtcaccacccgctgttcaaa
63   ccgataacgggttttcctccatggcgttgtgaagagcgtgcgattggggctatcagtga
123  tgtgatcttctctgtacaacacgcgaccagataacacctgtctggaactcaaactgcctg
183  cgcttggtcgttagcgcactatgacgcgggaattatgtgtacggtcataaacggttttgc
243  tgtacttatctcaccaagcaagaacttcacacggtttgtctcgctcgtgcgtggctcgcc
303  tggacgtagcgtcagtatccaagataaactaaccgtgtttttcacacgatgaggttttaa
363  ttaagtgacccctctcctcgtaattctgtcaatatttcttttgtttaaaactctgtata
423  ccagtgtctctgcagcctcctcacggaaaggtgttttaataatgagataatgattgagat
483  gaaacagtgagcggcggatcgttttgtggaaggtctatactcgatcaaagggcgaggac
543  gcgtttctggttttagggataccaaggtagttcatcaactgatagcaccagttcacgaaa
603  caggatcgagctgcttctcactcacaacgaacgttgctgtaaaggaaaactctcatcaaa
663  cattacggttactcttcggtcgaccaagccttcgtctgtcggactttaagccgttaaagt
723  gtgtctgcctgactagacgttgaaaccagtcgtccatagagcagcagcctctatcaaccc
783  aacacattttgtgctgatcgaggtaagagtgacgcgtagcttttgactcgtatcaagact
843  acacgagcagattacggcgttaagttgaccgttgtttttattagttgtcgagtggaaga
903  gctgacctatttttggtgtaccctcgagaccatagcgccatctgttgtccatgacattt
963  ggaatgactaatagctgcgaattaattaaaccgtaattggatcaactgtaatattcatca
1023 ttgtcaaaccgggatcttgcagacagttttgtttttttactttgaggaacaaaaccttcg
1083 gcacagcgtggcaagtggagcattgtttgtgcaaaaatggcgtccgtcggcaattctacc
                                          M  A  S  V  G  N  S  T    8
1143 caggagccgtttgcggacttgtatggtgattctgattggatctactctgtgaccgggtcc
      Q  E  P  F  A  D  L  Y  G  D  S  D  W  I  Y  S  V  T  G  S   28
1203 ttttcgtacattgtttgcgtcatcggactgctggcgaacacctgggtcatctgcgttttg
      F  S  Y  I  V  C  V  I  G  L  L  A  N  T  W  V  I  C  V  L   48
1263 ctatgcaagctcggactaaagaccgctggtaatatgtacatcctcaacctggccattgcc
      L  C  K  L  G  L  K  T  A  G  N  M  Y  I  L  N  L  A  I  A   68
1323 gatgacctttcttagctggtcttgccctccaggctgcctaccagatcacggaggtatgg
      D  D  L  F  L  A  G  L  A  L  Q  A  A  Y  Q  I  T  E  V  W   88
1383 cacttcggggagtttctatgccgtgccgcgctggcgttcgacggtctgaatatgtacgcg
      H  F  G  E  F  L  C  R  A  A  L  A  F  D  G  L  N  M  Y  A  108
1443 agtgccttcttcgtgacggctttgagcatcgagaggtacatggctgtcagccggtccagc
      S  A  F  F  V  T  A  L  S  I  E  R  Y  M  A  V  S  R  S  S  128
1503 cgggcccggctccatcgcaaacggcgccaggctgctgctgttagcgtcatcatctgggtg
      R  A  R  L  H  R  K  R  R  Q  A  A  A  V  S  V  I  I  W  V  148
1563 atttctatactagccgccattccgacgcttctagcaagttactacatcaccaacacgagg
      I  S  I  L  A  A  I  P  T  L  L  A  S  Y  Y  I  T  N  T  R  168
1623 gaagactacatctgcatcacgacgttctcaaacttcggcgaaccgggcgttgagttctgg
      E  D  Y  I  C  I  T  T  F  S  N  F  G  E  P  G  V  E  F  W  188
1683 aaccaggcgttcatcacctacaacttcgttctcggactctgcgtgccgctactggtaagc
      N  Q  A  F  I  T  Y  N  F  V  L  G  L  C  V  P  L  L  V  S  208
1743 tgcttctgctacggtatgctgattgttcaaatgagacaagtgtcaatgcggaatgacgcc
      C  F  C  Y  G  M  L  I  V  Q  M  R  Q  V  S  M  R  N  D  A  228
1803 ggtgctgacgtcaaacgcgtcacgcgtatcgtcaccggagtggtcgtagtcttctttctg
      G  A  D  V  K  R  V  T  R  I  V  T  G  V  V  V  V  F  F  L  248
1863 tgctgggcgccgttctatctagtacgaatgatactagtctataacccaagcctcatgtac
      C  W  A  P  F  Y  L  V  R  M  I  L  V  Y  N  P  S  L  M  Y  268
1923 tggagtggtaaaccatttgtgttcgagcttagtctttgcttcacgtatataaacagttgt
      W  S  G  K  P  F  V  F  E  L  S  L  C  F  T  Y  I  N  S  C  288
1983 gtcaatcctttatttacgcgctgattagtgagaagttcagggagaacttaccgtgtttg
      V  N  P  F  I  Y  A  L  I  S  E  K  F  R  E  N  L  P  C  L  308
2043 gcactccggggtcgcggatacatcagcgggaaaagaactgccgtctgtgaccggaactcg
      A  L  R  G  R  G  Y  I  S  G  K  R  T  A  V  C  D  R  N  S  328
2103 acggcactgacggacatccatcggaactctgtcataacaaacaatgattctagttagata
      T  A  L  T  D  I  H  R  N  S  V  I  T  N  N  D  S  S  *     346
2163 atctactctctgattttgaagagctgtgaacttcacgtgaacttcgtgaaatgttttcgc
2223 tgaagagatttttgcgttgtccggattaactttagctggttcgcggtacagtgcaatgc
2283 tgcgctggtaacagtggcattatgtctgcctagtgaacataactctatgaaaagacccgc
2343 ctgtgccatggtaccaatgttaacgggttatcatgaagacgagagatcggaagagcgtc
```

Supplementary Figure 6. ***Asterias rubens*** **SS-type receptor 1 (ArSSR1).** The nucleotide sequence (lowercase, numbered on the left) encoding ArSSR1 (uppercase, numbered on the right) is shown. The asterisk shows the position of the stop codon. This sequence was determined from contig 1120385 of an *A. rubens* radial nerve cord transcriptome and has been deposited in GenBank under accession number MN251831. A cDNA encoding ArSSR1 was amplified by PCR using specific primers (underlined; and with a partial Kozak sequence (ACC) added before the ATG start codon), cloned into the expression vector pcDNA 3.1(+) and then sequenced. The cDNA and contig sequences were identical in all but three nucleotides, which include a synonymous substitution (highlighted in blue) and two non-synonymous substitutions (highlighted in purple).

```
1                           aattttcctgcatatgtccacatgcaccttctaaagtgaaac
43   cgagttgggtgaactgacttaaaacttcaactgtgagttgcatcgtcgattgcgagaaaa
103  cgcagaatgagtatacattgaaacatccataggaacatcttggagtatacattgaaacat
163  ccataggaacatcttggagtattttgtacgaggatgagttcctaactcagtgttttactg
223  gtgagactaatttacaaaccaattttgcattcactcgtccagttgaccattggagctaaa
283  atggagccaactgtaacagaaacctatacatctttgtatccgatgaacgtcacagacttc
      M  E  P  T  V  T  E  T  Y  T  S  L  Y  P  M  N  V  T  D  F   20
343  tcgactctaaacctaacggaggaggaggaggaggaggggtaacttttctgatattcag
      S  T  L  N  L  T  E  E  E  E  E  E  E  G  N  F  S  D  I  Q   40
403  ctagccggtacaatcatcggttctctttatatgattatcaccatagttggtatccttggc
      L  A  G  T  I  I  G  S  L  Y  M  I  I  T  I  V  G  I  L  G   60
463  aatggcactgtaatctacgtagtgttaaggttcgctaagatgaaaacggtcacaaactgt
      N  G  T  V  I  Y  V  V  L  R  F  A  K  M  K  T  V  T  N  C   80
523  tacattcttaacctggccgtagctgatgcagtctttgtgaccttcttgacattaatggcc
      Y  I  L  N  L  A  V  A  D  A  V  F  V  T  F  L  T  L  M  A  100
583  gtatccaactttatggaaacctactggattttcggtgctttcttgtgtaaagtttacttt
      V  S  N  F  M  E  T  Y  W  I  F  G  A  F  L  C  K  V  Y  F  120
643  ggtatcgacatgttcaacatggtcatcagtgtatggtgtttgacagctatgagtgtggat
      G  I  D  M  F  N  M  V  I  S  V  W  C  L  T  A  M  S  V  D  140
703  cgctatgtagctgtttgccatgccatgaagtctcgaagcttcaggaatctaccaatcgct
      R  Y  V  A  V  C  H  A  M  K  S  R  S  F  R  N  L  P  I  A  160
763  acagctatcaatgcttccacatggttgctgtccattctggctgcaattccgttcgtctat
      T  A  I  N  A  S  T  W  L  L  S  I  L  A  A  I  P  F  V  Y  180
823  ttcgccaagttagaaccaggcagcggtaactacgatgtgtgttatctttacttcgaaccc
      F  A  K  L  E  P  G  S  G  N  Y  D  V  C  Y  L  Y  F  E  P  200
883  gatcgggtcaaaacgtcgagccagataatggccatgtgtgtgtttatattcaactttgtc
      D  R  V  K  T  S  S  Q  I  M  A  M  C  V  F  I  F  N  F  V  220
943  attccgttgactgtcatcatagtctgctacgcaagcatcaccatgcggctacgagatatg
      I  P  L  T  V  I  I  V  C  Y  A  S  I  T  M  R  L  R  D  M  240
1003 aacaaaaagacaggcaaaccggagaaatcacgaaaggtaaatcgcctggtcctgattgtc
      N  K  K  T  G  K  P  E  K  S  R  K  V  N  R  L  V  L  I  V  260
1063 gtgatcaccttcgtcatctgctgggctcctttctacattgtaaagattctcttcgtcttt
      V  I  T  F  V  I  C  W  A  P  F  Y  I  V  K  I  L  F  V  F  280
1123 gtggataccttcaggagatggaacaagacttggatcctctcggacataaccatgtcgttg
      V  D  T  F  R  R  W  N  K  T  W  I  L  S  D  I  T  M  S  L  300
1183 acatacatcaacagctgcgccaatcctttcctctacgcgttctttagcgacaacttcaga
      T  Y  I  N  S  C  A  N  P  F  L  Y  A  F  F  S  D  N  F  R  320
1243 aagagcttccggaaggcgtggttgtgccactcaagaaaccaggctgaggtgagccagacc
      K  S  F  R  K  A  W  L  C  H  S  R  N  Q  A  E  V  S  Q  T  340
1303 tcctatgcgagcaggtggaagttcggtaagagcggaggcaagggcaagtttaacaaaaag
      S  Y  A  S  R  W  K  F  G  K  S  G  G  K  G  K  F  N  K  K  360
1363 gggaagcggtttcgtttacacgatgatgagggagacgacactaatgtttccgggtataac
      G  K  R  F  R  L  H  D  D  E  G  D  D  T  N  V  S  G  Y  N  380
1423 aaccagtacccgctcacagctactgcagtgacttctgtcgtttcggaatcgtcctaccca
      N  Q  Y  P  L  T  A  T  A  V  T  S  V  V  S  E  S  S  Y  P  400
1483 aggaatggtgagatgagtcaagttaatttggataaacaagccgtagcgaaggcataaacg
      R  N  G  E  M  S  Q  V  N  L  D  K  Q  A  V  A  K  A  *     418
1543 gctgtgctgtaaagccaagtcattctacaaaattagtattaaaaagccttataaaatcgt
1603 acacctagaaaagaaagctttcgtacacaaagttgcgaaaaaaaaattcaacttcttttg
1663 acaatatagttactaactcaatcagtttgtttttataatttaacaaagtgtttttattaa
1723 aaatgtacccttttaaaatacactggacacatttggtaacaatcaattgagagctgttga
1783 tag
```

Supplementary Figure 7. ***Asterias rubens*** **SS-type receptor 2 (ArSSR2).** The nucleotide sequence (lowercase, numbered on the left) encoding ArSSR2 (uppercase, numbered on the right) is shown. The asterisk shows the position of the stop codon. This sequence was determined from contig 987179 (bases 1-381) and contig 1108044 (bases 362-1785) of an *A. rubens* radial nerve cord transcriptome and has been deposited in GenBank under accession number MN251832. The region of overlap between the two contigs is highlighted in green. A cDNA encoding ArSSR2 was amplified by PCR using specific primers (underlined; with a partial Kozak sequence (ACC) added before the ATG start codon), cloned into the expression vector pcDNA 3.1(+) and then sequenced. The cDNA and contig sequences were identical.

```
1                                                               t
2    gtccataaaagcagtaggccaactacggccgtcattcggaacctagccagctagcgagac
62   gggcgactcagacacggtcgcgggcggcaagaggggttccgtttttggcacgttccggc
122  tccgcaatttctcaaattaacctggattctaggatcaactgtcgatatcgacgtgccaca
182  atatgtgcatggatccatcgctgtcaagtgcagaatcgtactaccatgctgctatgtgtt
242  ttttgtaaacatcttgtcgagtgcactcagcatgcggagagaggagagttgccgccttta
302  ttcaccaagaaatatgtctctgttctaactgttttcgttcacacgtttatatgatttcct
362  gctcgatcaaatacgactgttcagttgttttgctctttaccaactcactggaaacggtct
422  gtctcaaaatgcacagaagacgacttacgttaagaacagaaactttgattatgaagagtt
482  aaaagtgtggtgagtcatcaaggaagtttggaatctcaaacactattttgcattcatact
542  ttaaaaggaaagttgagtacctttggcaaaatggattgcatggatacagcctcggatagt
                                 M  D  C  M  D  T  A  S  D  S   10
602  ttctccaccaattgttctttggagaattgtacagaagaggagactacgaagtcggtgacc
      F  S  T  N  C  S  L  E  N  C  T  E  E  E  T  T  K  S  V  T   30
662  caaattcgagattgggtggcgggaatttccttctgatcgtctgcataacgggtctgatt
      Q  I  R  D  W  V  A  G  I  F  L  L  I  V  C  I  T  G  L  I   50
722  ggcaacagcatggtcatctttgtggtgctacgctacgccaaaatgaaaaccgtcaccaat
      G  N  S  M  V  I  F  V  V  L  R  Y  A  K  M  K  T  V  T  N   70
782  ttgtacatcttgaatctggccatagccgatgacctgttcatgatcgcccttgtgtttctg
      L  Y  I  L  N  L  A  I  A  D  D  L  F  M  I  A  L  V  F  L   90
842  tctattgctaccctggctggtaacaattggatattcggccccgttctgtgccatgtggtc
      S  I  A  T  L  A  G  N  N  W  I  F  G  P  V  L  C  H  V  V  110
902  ttcgcaattgacggtctcaaccagtttaccagcgtgttttgcctgaccgccatgagcatg
      F  A  I  D  G  L  N  Q  F  T  S  V  F  C  L  T  A  M  S  M  130
962  gatcgctacgttgccgtatgccacgctcgaagggtccgcgggtatcggactcttcgcctg
      D  R  Y  V  A  V  C  H  A  R  R  V  R  G  Y  R  T  L  R  L  150
1022 gcaatctgcgtgaacttcggcgtatggttcctagcccttgccgctgccagcccgatgaat
      A  I  C  V  N  F  G  V  W  F  L  A  L  A  A  A  S  P  M  N  170
1082 atagtgacacggtacaccgtttacaacggtacaccactgtgttatttcaatttcgagtct
      I  V  T  R  Y  T  V  Y  N  G  T  P  L  C  Y  F  N  F  E  S  190
1142 gtattcggtaaagacgccgtcgtcataactcagaaactgttcatgatttacacgactttg
      V  F  G  K  D  A  V  V  I  T  Q  K  L  F  M  I  Y  T  T  L  210
1202 atgggacttgttattcctttagtcatcatttccgtgtgctattcgagcattgtggtgcgc
      M  G  L  V  I  P  L  V  I  I  S  V  C  Y  S  S  I  V  V  R  230
1262 ctcaagaaattaggaacccgcacgggaaagatgaagaaatcacgaaaggtaaaccgtcta
      L  K  K  L  G  T  R  T  G  K  M  K  K  S  R  K  V  N  R  L  250
1322 gtgtttttcgtagtgctcgccttcttcatgtgttggtggccgttttatgtatggcgaact
      V  F  F  V  V  L  A  F  F  M  C  W  W  P  F  Y  V  W  R  T  270
1382 atagtggtgtttgtaccgtgccttgggcaacagtggtttaatggcctcgtacttgacttt
      I  V  V  F  V  P  C  L  G  Q  Q  W  F  N  G  L  V  L  D  F  290
1442 acaatgtgtttgggttaccttaacagctgtgcaaatccactcctctacggattccttagc
      T  M  C  L  G  Y  L  N  S  C  A  N  P  L  L  Y  G  F  L  S  310
1502 gggaacttcaaaaagagtttccgtaaagtgtggaactgtacacaggaaacggagatacaa
      G  N  F  K  K  S  F  R  K  V  W  N  C  T  Q  E  T  E  I  Q  330
1562 aacggtcaacgccgtgccccggcccatcctatgtaatgtttgatcacaatcgatttatgt
      N  G  Q  R  R  A  P  A  H  P  M  *                          341
1622 tacggcaaaaagttgacttgtcttgataatatatctcgccaagacatcttattttgtgga
1682 ataagtcgagttggcatgatactgaatctcaccaagacgacccatttaattataagaatt
1742 cgacttgtctaaagagttaattcgcctaaacgtcatctaagaagccgactctgccgtcga
1802 tttcaccaaac
```

Supplementary Figure 8. ***Asterias rubens*** **SS-type receptor 3 (ArSSR3).** The nucleotide sequence (lowercase, numbered on the left) encoding ArSSR3 (uppercase, numbered on the right) is shown. The asterisk shows the position of the stop codon. This sequence was determined from scaffold 32375 of an *A. rubens* radial nerve cord transcriptome and has been deposited in GenBank under accession number MN251833. A cDNA encoding ArSSR3 was amplified by PCR using specific primers (underlined; with a partial Kozak sequence (ACC) added before the ATG start codon), cloned into the expression vector pcDNA 3.1(+) and then sequenced. The cDNA and scaffold sequences were identical.

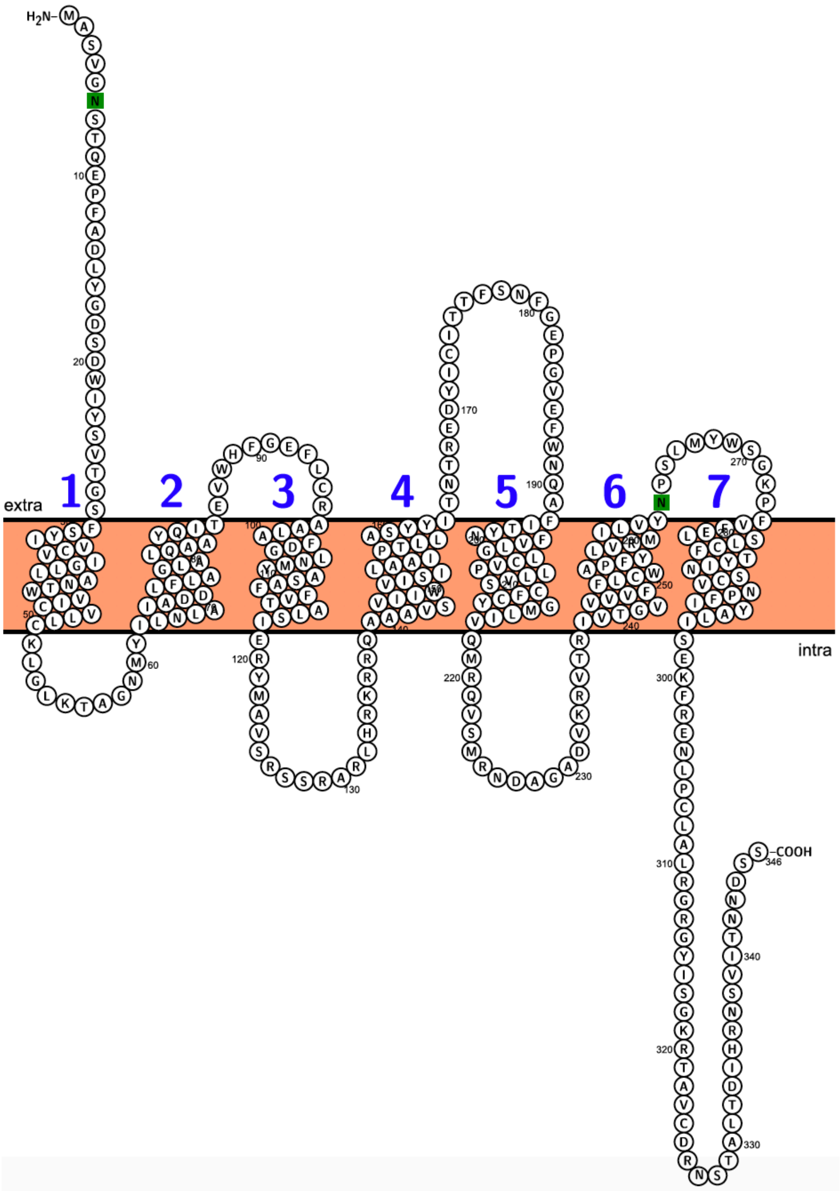

Supplementary Figure 9. Predicted topology of ArSSR1. Seven predicted membrane-spanning domains are numbered successively in blue and predicted N-glycosylation sites are highlighted in green. This figure was generated using Protter v1.0 (http://wlab.ethz.ch/protter/start/).

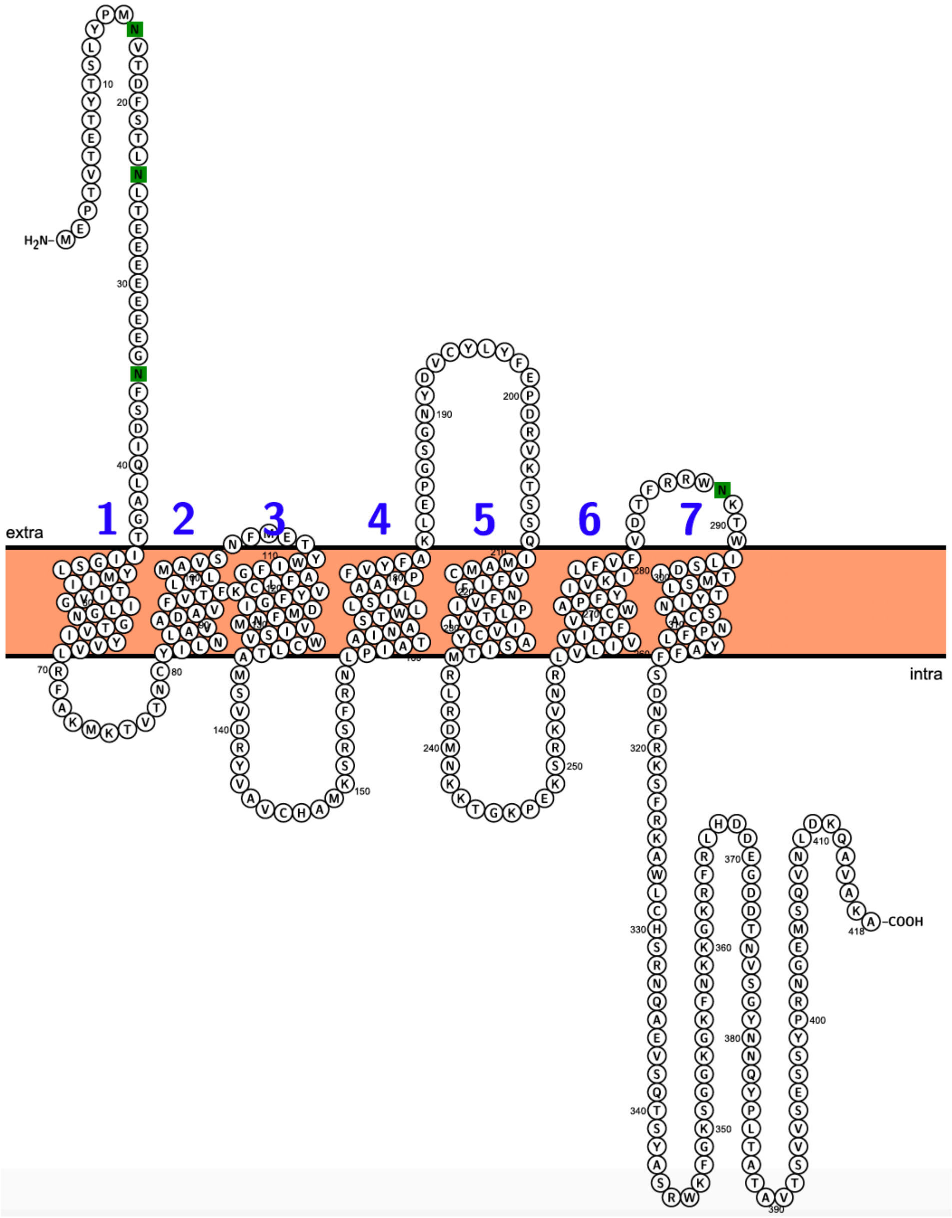

Supplementary Figure 10. Predicted topology of ArSSR2. Seven predicted membrane-spanning domains are numbered successively in blue and predicted N-glycosylation sites are highlighted in green. This figure was generated using Protter v1.0 (http://wlab.ethz.ch/protter/start/).

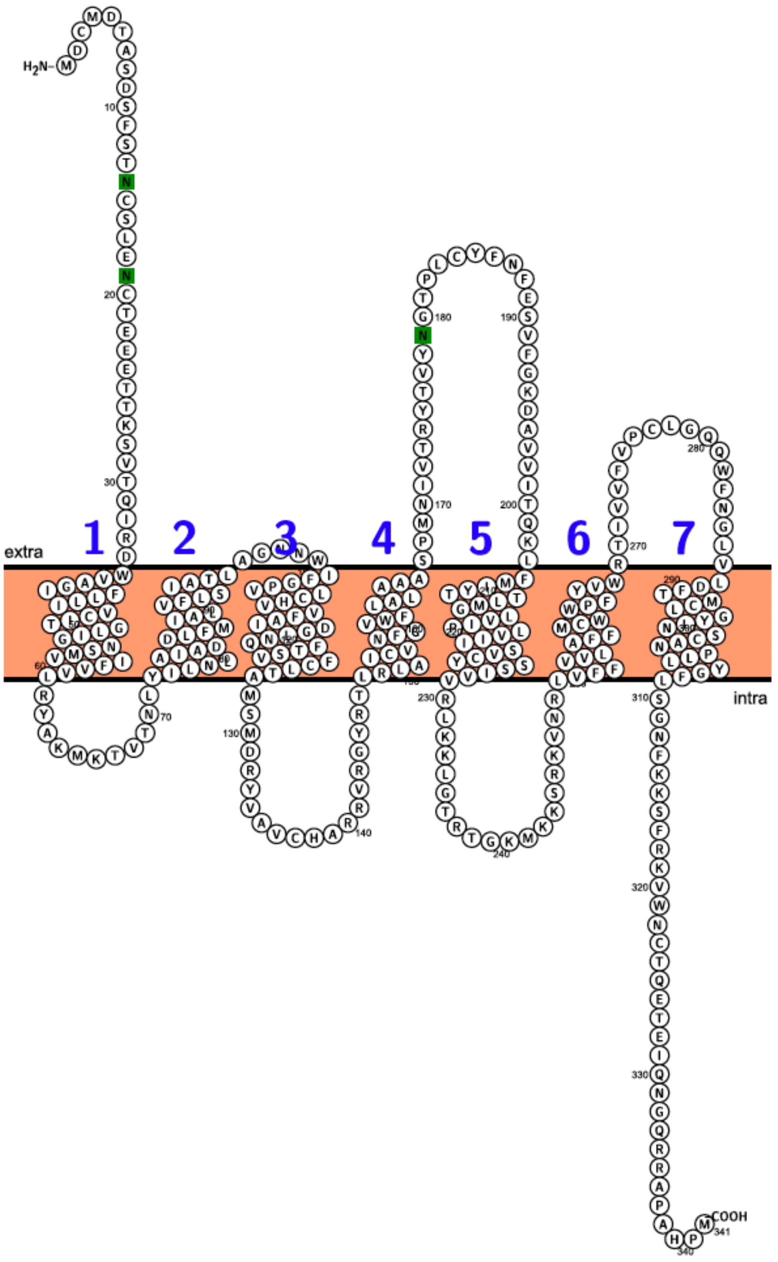

Supplementary Figure 11. Predicted topology of ArSSR3. Seven predicted membrane-spanning domains are numbered successively in blue and predicted N-glycosylation sites are highlighted in green. This figure was generated using Protter v1.0 (http://wlab.ethz.ch/protter/start/).

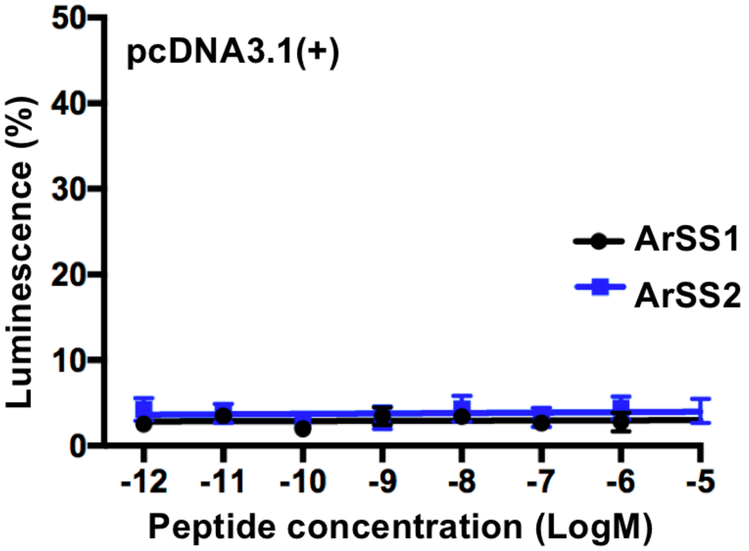

Supplementary Figure 12. ArSS1 and ArSS2 do not trigger luminscence in CHO-K1 cells transfected with an empty pcDNA3.1(+) vector. The graphs show that when tested at concentrations ranging from 10^{-12} and 10^{-5} M neither ArSS1 (black) nor ArSS2 (blue) trigger generation of luminescence above background levels in CHO-K1 cells stably expressing the calcium-sensitive bioluminescent GFP-aequorin fusion protein G5A. 0.1 % Triton X-100 was used as a positive control (100% activation) to normalize the luminescence values obtained from each well. These control experiments demonstrate that luminescence induced by ArSS2 in CHO-K1 cells transfected with the *A. rubens* SS/ASTC-type receptors ArSSR1-3 (Figure 4) is attributable to the transfected receptors and not receptors endogenously expressed in CHO-K1 cells.

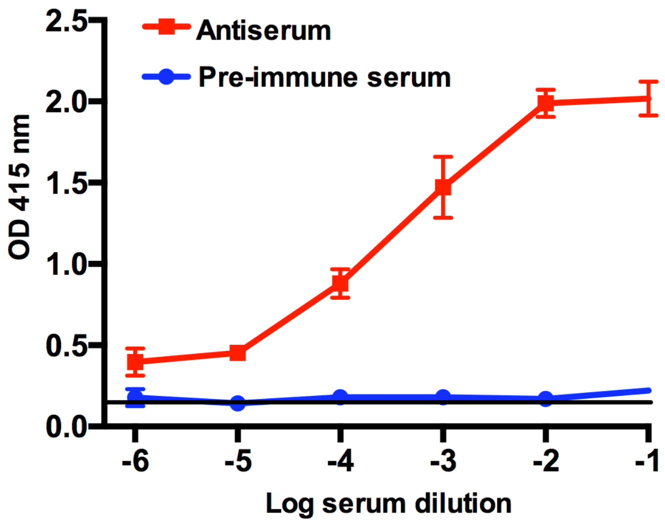

Supplementary Figure 13. Characterization of rabbit antiserum to ArSS2 using an enzyme-linked immunosorbent assay (ELISA). Incubation of antiserum (red) and pre-immune serum (blue) at dilutions between 10^{-6} and 10^{-1} with 0.1 nmol of antigen peptide (ArSS2) per well reveals no immunoreaction with pre-immune serum, whereas with the antiserum the antigen is detected at above the background optical density (black line) with dilutions from 10^{-6} to 10^{-1}.

Supplementary Table 1. Sequences of primers used for PCR cloning of cDNAs encoding *A. rubens* somatostatin-type precursors (ArSSP1, ArSSP2) and somatostatin-type receptors (ArSSR1-3)

**Transcript name**	**Primers (5'-3')**
**ArSSP1**	Forward primer: GAAACTCTACTTCTTTTAAACACC<br>Reverse primer: ATAGGCTGGGTGAACTCTAA
**ArSSP2**	Forward primer: ATGAATGCCTCGCCTATGATGAG<br>Reverse primer: CTAGCAGTCAACCAGTCCTCTG
**ArSSR1**	Forward primer: ACCATGGCGTCCGTCGGCAAT<br>Reverse primer: CTAACTAGAATCATTGTTTGTTATGACAGAGTTCCG
**ArSSR2**	Forward primer: ACCATGGAGCCAACTGTAACAGAAACCTAT<br>Reverse primer: TTATGCCTTCGCTACGGCTTGTTT
**ArSSR3**	Forward primer: ACCATGGATTGCATGGATAC<br>Reverse primer: TTACATAGGATGGGCCGGGG
**M13 primers**	Forward primer: GTAAAACGACGGCCAGTG<br>Reverse primer: GGAAACAGCTATGACCATG

Supplementary Table 2. Accession numbers of the precursor sequences used for the neuropeptide alignment in Figure 1

**Species name**	**Neuropeptide precursor name**	**Accession number or reference**
Homo sapiens	Somatostatin	NP_001039.1
Homo sapiens	Cortistatin	NP_001293.3
Homo sapiens	Urotensin II	NP_006777.1
Homo sapiens	Urotensin II related peptide	BAC98929.1
Branchiostoma floridae	Somatostatin	Semmens et al. 2016
Asterias rubens	Somatostatin2	MN257487
Acanthaster planci	Somatostatin2	gbr.24.36.t1 *
Ophionotus victoriae	Somatostatin2	Zandawala et al. 2017
Strongylocentrotus purpuratus	Somatostatin2	Zandawala et al. 2017
Apostichopus japonicus	Somatostatin2	Zandawala et al. 2017
Asterias rubens	Somatostatin1	ALJ99950.1
Acanthaster planci	Somatostatin1	XP_022111848.1
Ophionotus victoriae	Somatostatin1	XP_001176809.1
Strongylocentrotus purpuratus	Somatostatin1	Zandawala et al. 2017
Apostichopus japonicus	Somatostatin1	AWU78771.1
Drosophila melanogaster	Allatostatin C	NP_001162948.1
Drosophila melanogaster	Allatostatin double C	NP_609483.2
Caenorhabditis elegans	Allatostatin C	NP_491699.2
Capitella teleta	Allatostatin C	Mirabeau et al. 2013
Lottia gigantea	Allatostatin C	Mirabeau et al. 2013

* Note that the accession number gbr.24.36.t1 is from the database *Acanthaster planci* ver. 1.0 (Hall et al., 2017). https://marinegenomics.oist.jp/cots/viewer/info?project_id=46

Supplementary Table 3. Accession numbers of the sequences used for the gene structure analysis in Figure 2.

Species name	Gene name	mRNA	Protein	Genome
Homo sapiens	Somatostatin	NM_001048.4	NP_001039.1	Whole genome accessible with NCBI SPLIGN tool
Homo sapiens	Cortistatin	NM_001302.5	NP_001293.3	Whole genome accessible with NCBI SPLIGN tool
Homo sapiens	Urotensin II	NM_006786.4	NP_006777.1	Whole genome accessible with NCBI SPLIGN tool
Homo sapiens	Urotensin II related peptide	AB116021.1	BAC98929.1	Whole genome accessible with NCBI SPLIGN tool
Acanthaster planci	SS2	gbr.24.36.t1	gbr.24.36.t1	NW_019091382.1
Strongylocentrotus purpuratus	SS2	XM_001176669.3	XP_001176669.1	Whole genome accessible with NCBI SPLIGN tool
Acanthaster planci	SS1	XM_022256156.1	XP_022111848.1	NW_019091362.1
Strongylocentrotus purpuratus	SS1	XM_001176669	XP_001176809.1	Whole genome accessible with NCBI SPLIGN tool
Mizuhopecten yessoensis	Allatostatin C	XM_021500718.1	XP_021356393.1	NW_018403697.1
Caenorhabditis elegans	Allatostatin C	NM_059298.5	NP_491699.2	NC_003279.8
Drosophila melanogaster	Allatostatin C	NM_001169477.3	NP_001162948.1	Whole genome accessible with NCBI SPLIGN tool

Supplementary Table 4. Accession numbers and references for the amino acid sequences of the receptors used for the phylogenetic analysis in Figure 3.

**Receptor name (reference)**	**Species**	**GenBank Acession Number**
Pmar-OpioidR mu1c	*Petromyzon marinus*	ALF99986.1
Pmar-OpioidR mu1b	*Petromyzon marinus*	ALF99985.1
Pmar-OpioidR mu1a	*Petromyzon marinus*	ALF99984.1
Hsap-OpioidR mu (Pert et al., 1973)	*Homo sapiens*	NP_000905.3
Hsap-OpioidR delta (Portoghese et al., 1988)	*Homo sapiens*	NP_000902.3
Hsap-OpioidR kappa (Portoghese et al., 1987)	*Homo sapiens*	NP_000903.2
Bflo-OpoidR1	*Branchiostoma floridae*	XP_002606117.1
Bflo-OpoidR2	*Branchiostoma floridae*	XP_002590140.1
Pmar-SSR1	*Petromyzon marinus*	ALF99958.1
Hsap-SSR1 (Yamada et al., 1992a)	*Homo sapiens*	NP_001040.1
Hsap-SSR4 (Demchyshyn et al., 1993)	*Homo sapiens*	NP_001043.2
Hsap-SSR2 (Yamada et al., 1992a)	*Homo sapiens*	NP_001041.1
Hsap-SSR3 (Yamada et al., 1992b)	*Homo sapiens*	NP_001042.1
Hsap-SSR5 (Panetta et al., 1994)	*Homo sapiens*	NP_001044.1
Bflo-SSR	*Branchiostoma floridae*	XP_002590145.1
Arub-SSR1	*Asterias rubens*	MN251831
Apla-SSR1	*Acanthaster planci*	XP_022085547.1
Ovic-SSR	*Ophionotus victoriae*	See below for sequence *
Arub-SSR3	*Asterias rubens*	MN251833
Apla-SSR1	*Acanthaster planci*	XP_022080909.1
Spur-SSR	*Strongylocentrotus purpuratus*	XP_011665623.1
Arub-SSR2	*Asterias rubens*	MN251832
Apla-SSR2	*Acanthaster planci*	XP_022093677.1
Ajap-SSR	*Apostichopus japonicus*	PIK55903.1
Acal-ASTCR	*Aplysia californica*	XP_005095139.1
Lgig-ASTCR	*Lottia gigantea*	XP_009065270.1
Cgig-ASTCR	*Crassostrea gigas*	XP_011429560.1
Hrob-ASTCR	*Helobdella robusta*	XP_009020674.1
Ctel-ASTCR	*Capitella teleta*	ELT88806.1
Pdum-ASTCR (Bauknecht and Jekely, 2015)	*Platynereis dumerilii*	AKQ62999.1
Cele-ASTCR1	*Caenorhabditis elegans*	NP_001355490.1

Cele-ASTCR2	*Caenorhabditis elegans*	NP_503056.4
Cele-ASTCR3	*Caenorhabditis elegans*	NP_510833.3
Dpul-ASTCR	*Daphnia pulex*	EFX72687.1
Dmel-ASTCR2 (Kreienkamp et al., 2002)	*Drosophila melanogaster*	NP_001027135.2
Dmel-ASTCR1 (Kreienkamp et al., 2002)	*Drosophila melanogaster*	NP_649040.2
Amel-ASTCR	*Apis mellifera*	XP_006560939.1
Tcas-ASTCR	*Tribolium castaneum*	NP_001280521.1
Pmar-MCHR	*Petromyzon marinus*	ALF99968.1
Hsap-MCHR1 (An et al., 2001)	*Homo sapiens*	NP_005288.3
Hsap-MCHR2 (Sailer et al., 2001)	*Homo sapiens*	NP_001035269.1
Bflo-MCHR	*Branchiostoma floridae*	XP_002595601.1
Skow-MCHR1	*Saccoglossus kowalevskii*	XP_002740034.1
Skow-MCHR2	*Saccoglossus kowalevskii*	XP_002740035.2
Arub-MCHR1	*Asterias rubens*	See below for sequence *
Apla-MCHR1	*Acanthaster planci*	XP_022079320.1
Ovic-MCHR	*Ophionotus victoriae*	See below for sequence *
Spur-MCHR1	*Strongylocentrotus purpuratus*	XP_011667299.1
Arub-MCHR2	*Asterias rubens*	See below for sequence *
Apla-MCHR2	*Acanthaster planci*	XP_022096004.1
Spur-MCHR2	*Strongylocentrotus purpuratus*	XP_011670235.1
Ajap-MCHR	*Apostichopus japonicus*	PIK43705.1
Hsap-UTS2R (Liu et al., 1999)	*Homo sapiens*	NP_061822.1
Skow-UTS2R	*Saccoglossus kowalevskii*	XP_00273A245.1
Spur-UTS2R1	*Strongylocentrotus purpuratus*	XP_003730470.1
Spur-UTS2R2	*Strongylocentrotus purpuratus*	XP_003728476.1
Hsap-Galanin R3	*Homo sapiens*	NP_003605.1
Hsap-Galanin R2	*Homo sapiens*	NP_003848.1
Hsap-Galanin R1	*Homo sapiens*	NP_001471.2
Apla-Galanin R1	*Acanthaster planci*	XP_022095449.1
Skow-Galanin R	*Saccoglossus kowalevskii*	XP_002731810.1
Acal-ASTAR	*Aplysia californica*	XP_005097945.1
Cgig-ASTAR	*Crassostrea gigas*	XP_011447370.1
Pdum-ASTAR1	*Platynereis dumerilii*	AKQ63073.1
Pdum-GPR5	*Platynereis dumerilii*	AKQ63000.1
Ctel-ASTAR	*Capitella teleta*	ELU06086.1
Pman-Kiss-1R	*Peromyscus maniculatus bairdii*	XP_006978279.1
Mmus-Kiss-1R	*Mus musculus*	NP_444474.1

Hsap-GPR54	*Homo sapiens*	AAK83235.1
Shar-Kiss-1R	*Sarcophilus harrisii*	XP_012397698.2
Ctel-Kiss-1R	*Capitella teleta*	ELU15450.1
Pdum-GPR54	*Platynereis dumerilii*	AKQ63059.1
Lgig-Kiss-1R	*Lottia gigantea*	XP_009067091.1
Cgig-GPR54-isoform1	*Crassostrea gigas*	XP_011416956.1
Cgig-GPR54-isoform2	*Crassostrea gigas*	XP_011416957.1
Cgig-GPR54	*Crassostrea gigas*	XP_011431120.1

*** Sequences of receptor proteins without accession numbers:**

O. victoriae SSR
MYILMLAIADDLFLCSLIFQAASIITFSWPFGEVLCYAVMAIDGLNMYASAFFVTSMSIERY
LAVRGSTRARIHRGRRKVVVVSIIVWIVALIAATPSVIMTEYYSHPTQGISCSLNMAYLTGG
NQSDQDAHTLGARIFITYNFTLNFFFPLTVTSLCYGRLINQMKQVSIRNASGTSADLGRVAR
VVTGVVVVFFICWSPFYISRLLMAYFPKLNNWYGMAYVFELTLCFSYANSCLNPIIYALIND
KFRENLPSLPCCPKNEGYSKGQRKHQRMTSIRSTAQTDVHRSADYH

A. rubens MCHR1
MDKSTSIMLTTAGYSDILTPMFCANCTTPAGHPQDNSFLMSLQQISFIVTCILGTVGLLCNG
FIILILMRFPNMKTLANYFILNLALADFLFMISFLFLGHQLRVNHWVFGKFMCRLIVPYDAM
TQFLVIYFVMIMSIDRYFAICLPIKSMNFRTLRNGKIVCAMIWGVAILTTLPLWIYTEHTCV
NGWCTCLARVSSNDEDDPRWWIIYTIIIGFCVPLTIVCICYLLILQNLLTSNMQDSKNTLRR
AARRVAILVICIIIVFILCFLPFYVFQLVYSTIDAEEDASNALILIYIITWTLMYSHSIINP
IVYALVGENFRKNITFMFCKRSSRHVMYTRQSSMRTSSFRRTRNSLKSCNSNDPNGMRPHNG
YYVHLDTGQHRGAREDPV

A. rubens MCHR2
MEGNLHSDLSTQVSSAKMYSLTTQHPVYQGENNTDYWDNSSSDAISNATENIQSVLFQRFLA
PLIFGCISIVGIVANGLVIIVLLKYANMRTIPNVYILSLALMDFMFVLSLPMVGYQFCTNNW
PFGYFMCKLVPGIDGFNQFGSVNIMTMMSADRYVAVVYPLSSMRYRTKKTARIVCGVVFLIS
FILCTPSWYFMELKDGGYGLTYCIAKGPSWDKEGTLYTLYYACFGFLIPLAIIIVCYSTLLF
KILGSKLRIRSDGTGTAQRASKRVFILTVSVIVVFVVCWLPYNVVQLFHQFTTAEVSHAFSI
AYAVSSLWCYTNSCFNPIVYTFIGENFKKNVMHLCPFCTSPDDTKLDRYRSNRESSTYIRGG
TLRLHSVQHEAPASPPAMTTNATAITDATSFTEAPANTQTYTFSNNNTHNSCGKE

O. victoriae MCHR
MLTTSGYAEQHLFPNCSVDDPVPECYEEDNFQANVAKYFPPAIGVISGLGLLFNGFIIAVLL
RFTNMKTLANTFILNLALADFLFMISFIFLGHQMAYSQWVFGPYICRIIVPYDAMTQFTIIF
FLSTMSIDRYFAICLPFQSMSFRTLKSARIVSICVWIFAILVVLPVWLYSKEVQMPYFDNST
NETYWASVCMAGVAPNPEADTRWGIIYTMLIGFCLPLTAICICYLMIMYNLLNTPIQVAKNT
SRKAARRVAILVISIVIVFILCFLPFYVVQLILGNYGQQQPPKFMLIIYSVSWFLMYSHSII
NPIVYTIVGENFRQNLVRICRPRRKRGNATRQSSMRTSVTASKRTRNSCRSNYGTPETGHKL
PA

References

An, S., Cutler, G., Zhao, J. J., Huang, S. G., Tian, H., Li, W., Liang, L., Rich, M., Bakleh, A., Du, J. et al. (2001). Identification and characterization of a melanin-concentrating hormone receptor. *Proc Natl Acad Sci U S A* **98**, 7576-81.

Bauknecht, P. and Jekely, G. (2015). Large-Scale Combinatorial Deorphanization of Platynereis Neuropeptide GPCRs. *Cell Rep* **12**, 684-93.

Demchyshyn, L. L., Srikant, C. B., Sunahara, R. K., Kent, G., Seeman, P., Van Tol, H. H., Panetta, R., Patel, Y. C. and Niznik, H. B. (1993). Cloning and expression of a human somatostatin-14-selective receptor variant (somatostatin receptor 4) located on chromosome 20. *Mol Pharmacol* **43**, 894-901.

Hall, M. R., Kocot, K. M., Baughman, K. W., Fernandez-Valverde, S. L., Gauthier, M. E. A., Hatleberg, W. L., Krishnan, A., McDougall, C., Motti, C. A., Shoguchi, E. et al. (2017). The crown-of-thorns starfish genome as a guide for biocontrol of this coral reef pest. *Nature* **544**, 231-234.

Kreienkamp, H. J., Larusson, H. J., Witte, I., Roeder, T., Birgul, N., Honck, H. H., Harder, S., Ellinghausen, G., Buck, F. and Richter, D. (2002). Functional annotation of two orphan G-protein-coupled receptors, Drostar1 and -2, from Drosophila melanogaster and their ligands by reverse pharmacology. *J Biol Chem* **277**, 39937-43.

Liu, Q., Pong, S. S., Zeng, Z., Zhang, Q., Howard, A. D., Williams, D. L., Jr., Davidoff, M., Wang, R., Austin, C. P., McDonald, T. P. et al. (1999). Identification of urotensin II as the endogenous ligand for the orphan G-protein-coupled receptor GPR14. *Biochem Biophys Res Commun* **266**, 174-8.

Panetta, R., Greenwood, M. T., Warszynska, A., Demchyshyn, L. L., Day, R., Niznik, H. B., Srikant, C. B. and Patel, Y. C. (1994). Molecular cloning, functional characterization, and chromosomal localization of a human somatostatin receptor (somatostatin receptor type 5) with preferential affinity for somatostatin-28. *Mol Pharmacol* **45**, 417-27.

Pert, C. B., Pasternak, G. and Snyder, S. H. (1973). Opiate agonists and antagonists discriminated by receptor binding in brain. *Science* **182**, 1359-61.

Portoghese, P. S., Lipkowski, A. W. and Takemori, A. E. (1987). Binaltorphimine and nor-binaltorphimine, potent and selective kappa-opioid receptor antagonists. *Life Sci* **40**, 1287-92.

Portoghese, P. S., Sultana, M. and Takemori, A. E. (1988). Naltrindole, a highly selective and potent non-peptide delta opioid receptor antagonist. *Eur J Pharmacol* **146**, 185-6.

Sailer, A. W., Sano, H., Zeng, Z., McDonald, T. P., Pan, J., Pong, S. S., Feighner, S. D., Tan, C. P., Fukami, T., Iwaasa, H. et al. (2001). Identification and characterization of a second melanin-concentrating hormone receptor, MCH-2R. *Proc Natl Acad Sci U S A* **98**, 7564-9.

Yamada, Y., Post, S. R., Wang, K., Tager, H. S., Bell, G. I. and Seino, S. (1992a). Cloning and functional characterization of a family of human and mouse somatostatin receptors expressed in brain, gastrointestinal tract, and kidney. *Proc Natl Acad Sci U S A* **89**, 251-5.

Yamada, Y., Reisine, T., Law, S. F., Ihara, Y., Kubota, A., Kagimoto, S., Seino, M., Seino, Y., Bell, G. I. and Seino, S. (1992b). Somatostatin receptors, an expanding gene family: cloning and functional characterization of human SSTR3, a protein coupled to adenylyl cyclase. *Mol Endocrinol* **6**, 2136-42.
